# Global facial soft tissue thicknesses for craniofacial identification (2023): a review of 140 years of data since Welcker’s first study

**DOI:** 10.1007/s00414-023-03087-x

**Published:** 2023-10-07

**Authors:** Te Wai Pounamu T. Hona, Carl N. Stephan

**Affiliations:** Laboratory for Human Craniofacial and Skeletal Identification (HuCS-ID Lab), School of Biomedical Sciences, The University of Queensland, Brisbane, 4072 Australia

**Keywords:** Craniofacial identification, Facial soft tissue depth, Facial approximation, Skull, Face

## Abstract

This year (2023) marks 140 years since the first publication of a facial soft tissue thickness (FSTT) study. Since 1883, a total of 139 studies have been published, collectively tallying > 220,000 tissue thickness measurements of > 19,500 adults. In just the last 5-years, 33 FSTT studies have been conducted. Herein, we add these data (plus an additional 20 studies) to the 2018 T-Table to provide an update of > 81,000 new datapoints to the global tallied facial soft tissue depths table. In contrast to the original 2008 T-Table, some notable changes are as follows: increased FSTTs by 3 mm at infra second molar (ecm_2_–iM_2_ʹ), 2.5 mm at gonion (go–goʹ), 2 mm at mid-ramus (mr–mrʹ), and 1.5 mm at zygion (zy–zyʹ). Rolling grand means indicate that stable values have been attained for all nine median FSTT landmarks, while six out of nine bilateral landmarks continue to show ongoing fluctuations, indicating further data collection at these landmarks holds value. When used as point estimators for individuals with known values across 24 landmarks (i.e., C-Table data), the updated grand means produce slightly less estimation error than the 2018 T-Table means (3.5 mm versus 3.6 mm, respectively). Future efforts to produce less noisy datasets (i.e., reduce measurement and sampling errors as much as possible between studies) would be useful.

**Supplementary Information:**

The online version contains supplementary material available at 10.1007/s00414-023-03087-x.

## Introduction

Facial soft tissue thicknesses (FSTTs) form the quantitative basis of craniofacial identification techniques by providing a metric guide to the depth of the soft tissue envelope that overlies the skull [1–3]. In craniofacial superimposition, mean FSTT markers are placed at specific craniometric landmarks to help determine if the skull is a plausible fit to a once living person’s facial contours as recorded in a facial photograph [1]. If the skull is a good fit, the tissue depth markers should align to the skin surface in the superimposed facial photograph, with only negligible differences. In facial approximation, similar applies, however, instead of using an antemortem reference photograph, mean FSTTs are used as a guide to how much soft tissue should be added to the skull to approximate an individual’s face [2, 3]. This applies no matter which facial approximation method is used, including so-called “Russian,” “American,” and “Combination” methods, as all methods, including Gerasimov’s techniques, use mean FSTTs [3–7].

While FSTT means have been criticized because they represent average values [8, 9], these means have always served the intent of providing a *general indication* to what an individual’s true FSTT might be, rather than exact individualized point estimates free of any error [2, 10]. In other words, their goal is central tendency description of a sample, not estimation of precise values for single individuals. When FSTT means are employed as general guides in the craniofacial identification context, they are used together with a tolerance to account for sampling errors and individual variation. Both the standard error of the mean and the standard deviation provides practitioners leeway to modify mean FSTTs within statistically realistic ranges. These adjustments are often undertaken according to the robustness (or relief) of the skull [2, 3, 11, 12]. As facial approximations are undertaken in the blind, this maneuver tends to inject a degree of subjectivity into the methods. In contrast to FSTT means, regression approaches attempt to tailor estimations more precisely to individuals, typically via craniometric dimensions [8, 9, 13, 14]. However, just like means, these estimates still retain errors that can sometimes be large. In particular, the strength of craniometric correlations is weak, and generally have only been described for samples of small size, limiting their utility [9, 13, 14]. Consequently, arithmetic means continue to hold foundational value for craniofacial identification casework.

Over the past 140 years, >100 FSTT studies have been conducted on adults [15–17], with almost all FSTT studies following the same basic principles as established in 1883 by Welcker [18], whereby tissue thicknesses are measured from the skin surface to the most superficial aspect of the underlying bone at cephalometric landmarks [15]. Several measurement techniques have been used to acquire these FSTT data, including, solid-core needle puncture [19], lateral ‘plain film’ cephalograms [20], ultrasound (A- and B-mode) [21], computed tomography (CT) [22], cone beam computed tomography (CBCT) [23], and magnetic resonance imaging (MRI) [24, 25]. Despite the abundance of FSTT studies, individual study sample sizes tend to be small (*n* ≤ 40). This often applies when overarching large samples are employed (*n* > 100) as investigators commonly subdivide their samples into smaller subgroups, e.g., by sex, age, and/or ancestry [15, 26, 27].

The representativeness of small-sample FSTT studies is often problematic [28]; however, pooling multiple small-sample studies holds the potential to combat this limitation. One common hesitation to FSTT data pooling is that historically esteemed factors thought to be important for FSTTs (such as sex, ancestry, body position (supine/upright), and/or measurement method) are not separately retained. While categories are sometimes lost, pooled FSTTs tradeoff the often small differences for the benefits of larger sample sizes and increased representativeness under the Law of Large Numbers [16]. This is valuable in the FSTT context because there is no single recognized or agreed-upon gold standard method for FSTT measurement, such that investigators are currently using different methods that all produce slightly different results [29]. Subsequently, statistical noise exists in the FSTT dataset. Pooling the data averages out ‘noise’ either side of underlying ground truth values to produce more accurate means than those from single samples. In other words, under the central limit theorem, data pooling holds the advantage that the distribution of the sample means will increasingly approximate the normal distribution as more studies are included, producing a grand mean that converges on the underlying ground truth value [30, 31]. Since FSTT means are exclusively used as general guides to the typical value of soft tissue over the skull for many individuals, the loss of small differences like those attributable to sex (typically in the order of < 1 mm) with an ‘all-in’ data pool are, for the most part, inconsequential.

An additional benefit of data pooling is that the raw data are not required for the procedure; that is, grand means can be produced in a weighted fashion from just the central tendency statistic and the sample size. These principles drove the first calculation of the *tallied facial soft tissue depth tables* (commonly referred to as the T-Tables) in 2008 [15, 26]. The T-Tables represent three pooled data tables by age: 0–11 years, 12–17 years, and adults (≥ 18 years). Each 5-year period since their first production the newly published FSTT data have been added to the pooled data and the grand means updated (including weighted rolling means) [15–17, 26] (Table 1).

**Table 1:** Four iterations of the adult T-Tables in the last 15 years (2008–23)

T-Table	Contributing studies	No. studies per T-Table
2008 [15]	[3, 8, 18–22, 24, 32–72]; Fisher and Moorman (year unknown) cited in [73]; Köstler (1940), Bankowski (1958), Weining (1958), Weiber (1940); and Helmer (1984) cited in [74].	55
2013 [16]	2008 + [23, 25, 75–90]	55 + 18 new Total = 73
2018 [17]	2008 + 2013 + [91–103]	73 + 13 new Total = 86
2023	2008 + 2013 + 2018 + [9, 13, 104–154]*	86 + 53 new* Total = 139

*New study additions reported herein

Five years on from the latest iteration of the T-Table [17], additional sample-specific data from 4730 adults have been published, and FSTT data from a further 2978 adults have been extracted from the literature (pre-2018). Collectively, these new data (*n* > 7700) exceed the starting sample size of the 2008 T-Table (*n* ≈ 7400) and represent 39% of all FSTT data published to date, making an update to the adult T-Table worthwhile (Table 1). This 2023 T-Table update corresponds to the 140^th^ year anniversary since Welcker’s first FSTT study additionally making it very timely [18].

## Methods

Literature searches were conducted for all publications concerning facial soft tissue depth measurements using Scopus, PubMed, and Google Scholar, as well as traditional methods (e.g., reference list searches of relevant articles) to capture all relevant literature. Primary attention was awarded to studies published between 2018 and 2022; however, in the interest of thoroughness, studies from any year that had been missed in previous T-Table versions were also considered. As few new subadults studies were published, only adult studies that reported means and sample size for ≥ 3 landmarks with clear landmark definitions were evaluated. This resulted in 53 new FSTT studies contributing to the 2023 T-Table update, including 33 FSTT studies published between 2018 and 2022 [9, 13, 124–154], and an additional 20 FSTT studies published pre-2018 [104–123] (Table 1). These new data include all six main FSTT measurement methods used, so far, for data collection (Table 2).

**Table 2:** Data collection methods for studies included in the 2023 T-Table

Measurement method	Studies	*n*
CT	[22, 82, 84, 88, 89, 91, 92, 95, 97, 99, 100, 103, 105, 106, 110, 121, 129, 135, 143, 151, 154]	21
CBCT	[13, 23, 94, 113, 119, 131–134, 137, 144, 145, 152, 153]	14
Cephalogram	[20, 39, 42, 43, 45, 48, 52, 54, 55, 59, 60, 64–66, 69, 83, 87, 102, 104, 109, 112, 114–116, 118, 120, 125, 127, 130, 136, 139–142, 147–150]; Köstler (1940), Bankowski (1958), Weining (1958), Weiber (1940) in [74].	42
Ultrasound	[9, 21, 53, 58, 67, 68, 74, 85, 98, 101, 111, 117, 122, 124, 128]	15
MRI	[24, 25, 72, 78, 86, 90, 96, 126, 138]	9
Needle Puncture*	[8, 18, 19, 32–38, 40, 41, 46, 47, 49–51, 56, 57, 61, 70, 71, 75, 79–81, 93, 107, 108, 146]; Fisher and Moorman (year unknown) in [73].	31
Combination	Needle Puncture & Cephalograms: [3, 44] Ultrasound & Cephalograms: [62, 77] MRI & Needle Puncture: [63] MRI & CT: [123] CT & Tissue Cylinder Biopsy: [76]	7

*CT* computed tomography; *CBCT* cone beam computed tomography; *MRI* magnetic resonance imaging; *n* number of studies per measurement method

*Includes two studies that used non-conventional methods, including surgical blades [18] and clinical callipers [57]

Despite long-standing standardized nomenclature for craniofacial anthropometry [155], inconsistencies in landmark identification, description, and nomenclature were common across studies [156]. Therefore, landmark clarifications and reclassifications were required for data pooling. For example, some studies used standard landmark names, but non-standard definition(s) (e.g., gnathion name with menton definition), and proximally located landmarks were sometimes confused with one another (e.g., subspinale and mid-philtrum). As also found elsewhere [15, 156], imprecise lay vocabulary was sometimes used in place of technical terminology (e.g., ‘chin’ for either pogonion, gnathion, or menton landmarks depending on the study). In some instances, there was non-standard use of landmark abbreviations (e.g., description of only one landmark from a cephalometric landmark pair), or entirely new formulations for pre-existing landmarks (see [156] for more specific details). Additionally, reclassifications were made for landmarks where the original terms were inappropriate. For example, irrespective of other labels, studies clearly measuring the FSTT directly inferior to the mental symphysis were classed as menton, and FSTT measurements at the deepest point (in profile view) below the anterior nasal spine were classified as subnasale.

After the newly sourced data were added to the 2018 T-Table, weighted grand means and standard deviations were calculated to produce the updated 2023 T-Table values. These statistics were compared to both the 2008 T-Table (first iteration) [15] and the 2018 T-Table (last iteration) [17] to identify any changes. The convergence of FSTT data on stable rolling mean values was investigated for sex separated data by serially pooling study means in sequence of their publication date [16, 17]. As studies are pooled by their publication date, the weighted rolling mean data up to the end of 2017 is the same as the 2018 T-Table [17]—the new data here add to rolling means from 2018 to 2023.

The large volume of cross-sectional data within the 2023 T-Table facilitates the opportunity to examine the pooled data by the measurement method. While this analysis is cross-sectional, meaning different subjects are utilized for each measurement method, the large sample size is advantageous to provide insights into the potential effects of the different FSTT measurement methods. So that measurement method effects were salient, data were summed across landmarks to provide a general indicator of tissue volume across the face per measurement method [29]. The data were analyzed by the following: (1) the new studies identified in this review, (2) only 2018 T-Table studies, and (3) all studies combined. To enable the comparison by method, only those studies that reported data for the same landmarks could be used, so that no method was under-sampled in comparison to any other for any particular landmark. The selection of landmarks was therefore dictated by their commonality (highest *n* per Table 3). With regard to the median plane, five landmarks were used: glabella (g–gʹ), nasion (n–seʹ), rhinion (rhi–rhiʹ), supramentale (sm–smʹ), and pogonion (pg–pgʹ). With regard to bilateral positions, four landmarks were used: mid-supraorbital (mso–msoʹ), mid-infraorbital (mio–mioʹ), gonion (go–goʹ), and zygion (zy–zyʹ). Only studies that reported data for all five median landmarks or all four bilateral landmarks were included. Authors that reported FSTT data separately for different measurement methods in the same study (Table 2) were included for each method (total *n* = 146), resulting in a final sample of 93 studies for median landmark analysis and 59 studies for bilateral landmarks (Supplementary Table S1 and S2).

**Table 3:** Adult 2023 T-Table (≥ 18 Years)

	Total weighted mean (mm)	*n*	Weighted mean for *s* studies (mm)	*s* (mm)	*n*	Standard error of the mean (mm)
Median landmarks						
op–opʹ	6.0	2273	6.5	2.0	1992	0.0
v–vʹ	5.0	2272	5.0	1.5	1941	0.0
g–gʹ	5.5	15,937	5.5	1.0	14,400	0.0
n–seʹ	6.0	16,300	6.0	1.5	14,386	0.0
mn–mnʹ	4.5	3065	4.5	1.5	2711	0.0
rhi–rhiʹ	3.0	15,401	3.0	1.0	14,027	0.0
ss–snʹ	13.5	8897	13.5	3.5	8199	0.0
mp–mpʹ	11.5	10,717	11.0	2.5	9024	0.0
pr–lsʹ	12.0	14,894	12.0	3.0	13,764	0.0
id–liʹ	13.5	14,425	13.5	3.0	13,316	0.0
sm–smʹ	11.0	15,238	11.0	2.0	13,774	0.0
pg–pgʹ	11.0	17,075	11.0	2.5	14,861	0.0
gn–gnʹ	7.5	3259	8.0	2.5	2945	0.0
me–meʹ	7.0	13,583	7.0	2.5	12,394	0.0
Bilateral landmarks						
mso–msoʹ	7.0	8966	7.0	2.0	8337	0.0
mio–mioʹ	6.5	8966	7.0	3.0	8349	0.0
ac–acʹ	10.0	3417	10.0	3.0	3167	0.1
go–goʹ	12.5	9827	13.0	6.0	8739	0.1
zy–zyʹ	7.5	10,399	7.5	3.0	9089	0.0
sC–sCʹ	10.5	6439	10.5	2.5	6314	0.0
iC–iCʹ	11.0	3904	10.5	2.5	3777	0.0
ecm^2^–sM^2^ʹ	26.0	6203	26.5	7.0	5770	0.1
ecm_2_–iM_2_ʹ	22.0	4756	22.5	6.5	4323	0.1
mr–mrʹ	19.5	6344	19.5	5.0	6023	0.1
mmb–mmbʹ	11.0	4573	11.0	4.0	4186	0.1

Soft tissue depth values have been rounded to the nearest 0.5 mm. Measurement landmarks are based on [15]

*Total weighted mean* weighted mean across all studies in the literature reporting a soft tissue depth mean for the corresponding landmark, *n* sample size used to calculate each weighted mean, *s* weighted standard deviation, *Weighted mean for s studies* weighted means for studies that reported standard deviations

Standard error of the mean values has been rounded to the nearest 0.1 mm

The standard error of the 2023 means were calculated from the standard deviations and sample sizes (*s*/√*n*), while the point estimation accuracy for individuals with known FSTT values was tested following [27] using standard errors of the estimate, the v2018.1 C-Table and the TDValidator script [29] (the last two available at CRANIOFACIALidentification.com). As the 2023 data concern only adults, 511 individuals aged ≤ 17 years were removed from the v2018.1 C-Table prior to analysis. Any zero values (included by default for some missing entries in the public C-Table v2018.1) were also removed, so as not to interfere with the TDValidator script calculations. The C-Table test sample subsequently included known FSTTs from 1460 individuals, as contributed by 20 investigator teams from the following studies: [3, 8, 9, 18–20, 32, 34–39, 51, 56, 67, 71, 91, 95, 128].

## Results

The updated 2023 T-Table holds a total of 139 FSTT studies reporting 227,130 tissue thickness measurements for > 19,500 adults at 25 popularly measured landmarks (Table 3). This includes new data corresponding to 81,790 FSTT measurements from 7708 adults in addition to the last T-Table version [17]. The new data additions data represent 39% of the new total available T-Table dataset. The median landmark pogonion (pg–pg′) currently yields the largest sample size, with FSTT data from 17,075 adults (Table 3). The sample sizes for each landmark in this version are now large enough that the standard errors of the mean are approaching zero and thereby signal very high reliabilities (≤ 0.1 mm, Table 3).

### T-Table trends: 2008 versus 2023 data

Compared with the original 2008 T-Table, there have been some notable changes in FSTTs, namely, a 3 mm increase at infra second molar (ecm_2_–iM_2_ʹ), 2.5 mm increase at gonion (go–goʹ), 2 mm increase at mid-ramus (mr–mrʹ), and 1.5 mm increase at zygion (zy–zyʹ) (Table 4). Only small differences (≤ 1 mm) were evident at all other landmarks. Since 2008, the total sample size for over half of the median landmarks have increased by > 9000 individuals, while only four (of 11) bilateral landmarks have increased by > 5000 individuals (Table 4). The larger data availability for median landmarks is primarily underpinned by the increased number of lateral cephalometric studies that award most attention to the midline and bolster the reliability of these median landmark data (Table 2).

**Table 4:** Difference between the adult 2008 and 2018 T-Tables with the updated 2023 T-Table

	Δ total weighted mean (mm)		*n* increased by (Δ *n*)		Δ weighted means for *s* studies (mm)		Δ* s* (mm)		*n* increased by (Δ *n*)	
	Δ2023:08	Δ2023:18	Δ2023:08	Δ2023:18	Δ2023:08	Δ2023:18	Δ2023:08	Δ2023:18	Δ2023:08	Δ2023:18
Median landmarks										
op–opʹ	− 0.5	0.0	1121	0	0.0	0.0	− 0.5	0.0	1002	0
v–vʹ	0.0	0.0	1217	0	0.0	0.0	0.5	0.0	1156	0
g–gʹ	0.0	0.0	10,146	6061	0.0	0.0	0.0	0.0	9858	5961
n–seʹ	− 0.5	− 0.5	10,141	5967	0.0	0.0	0.0	0.0	9969	5867
mn–mnʹ	0.5	0.0	1793	993	0.5	0.0	0.5	0.0	1792	993
rhi–rhiʹ	0.0	0.0	9890	5848	0.0	0.0	0.0	0.0	9720	5748
ss–snʹ	0.5	0.5	7129	5614	**1.0**	0.5	0.5	0.5	7029	5514
mp–mpʹ	0.0	0.0	5209	1797	0.0	0.0	0.0	0.0	5069	1797
pr–lsʹ	0.5	0.0	9788	6409	0.5	0.0	0.0	0.0	9548	6309
id–liʹ	0.5	0.0	9539	6379	0.5	0.0	0.5	0.0	9299	6279
sm–smʹ	0.0	0.0	9446	5896	0.0	0.0	0.0	0.0	9277	5796
pg–pgʹ	− 0.5	0.0	10,289	6778	0.0	0.0	0.0	0.0	9970	6528
gn–gnʹ	− **1.0**	0.0	2714	1674	− 0.5	0.5	− 0.5	− 0.5	2564	1524
me–meʹ	0.0	− 0.5	9108	6068	0.0	0.0	0.0	0.0	8599	5818
Bilateral landmarks										
mso–msoʹ	**1.0**	0.0	6741	3327	**1.0**	0.5	0.5	0.0	6499	3227
mio–mioʹ	− 0.5	− 0.5	6668	3266	0.0	0.0	− 0.5	0.0	6439	3177
ac–acʹ	0.5	0.0	1906	858	0.5	0.0	**1.0**	0.5	1806	858
go–goʹ	**2.5**	0.5	5659	2799	**3.0**	0.5	0.0	− 0.5	5419	2699
zy–zyʹ	**1.5**	0.5	6009	2910	**1.5**	0.5	**2.0**	0.0	5544	2810
sC–sCʹ	**1.0**	0.0	3301	1136	**1.0**	0.5	0.5	0.0	3201	1136
iC–iCʹ	0.5	0.5	2720	1134	0.0	0.0	0.5	0.5	2620	1134
ecm^2^–sM^2^ʹ	0.5	**1.0**	4798	2419	0.5	**1.0**	**1.5**	**1.0**	4558	2319
ecm_2_–iM_2_ʹ	**3.0**	**2.0**	3412	1718	**3.0**	**2.0**	**2.0**	**1.5**	3172	1618
mr–mrʹ	**2.0**	0.5	3486	1417	**2.0**	0.5	**1.0**	0.5	3386	1417
mmb–mmbʹ	0.5	− **2.0**	3638	975	0.5	− **2.0**	− 0.5	0.5	3638	614

Values have been rounded to the nearest 0.5 mm. Differences between means exceeding 0.5 mm are bolded for ease of reference

### T-Table trends: 2018 versus 2023 data

Compared with the most recent T-Table version (2018), negligible differences in FSTTs (0–0.5 mm) were evident between all median landmarks and most bilateral landmarks, despite the sizeable addition of new data in the 2023 T-Table (Table 4). The exceptions were supra second molar (ecm^2^–sM^2^ʹ) and infra second molar (ecm_2_–iM_2_ʹ), which increased by 1 mm and 2 mm (respectively), and mid-mandibular boarder (mmb–mmbʹ), which decreased by 2 mm. Since 2018, the sample size for almost all median landmarks has risen by > 5000 individuals, while sample size for most bilateral landmarks have increased on average by approximately 2000 individuals (Table 4).

### Weighted rolling means

**Fig. 1:**
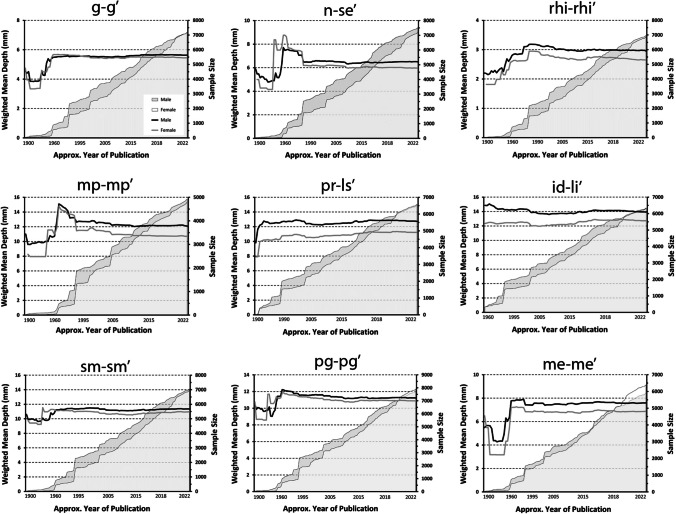
Weighted rolling means for adult data at nine common median landmarks. The rolling FSTT mean is shown on the principal axis (left), while the rolling total sample size is shown on the alternate axis (right)

**Fig. 2:**
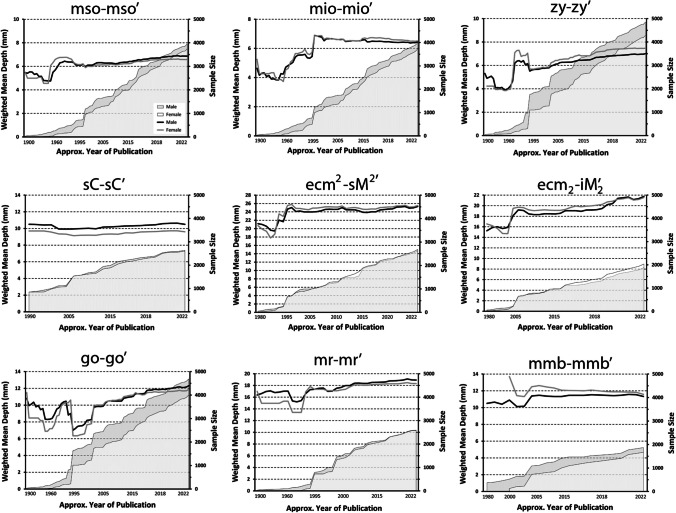
Weighted rolling means for adult data at nine common bilateral landmarks. The rolling FSTT mean is shown on the principal axis (left), while the rolling total sample size is shown on the alternate axis (right)

Line plots of weighted rolling means by year of data publication for all nine median landmarks illustrate convergence on stable mean values (Fig. 1). Early in the sequence when the sample sizes are small (< 2500 individuals), these rolling means exhibit increased movement and interweaving of sex specific means (Fig. 1). As the sample size increases, the means stabilize (underpinned by the Law of Large Numbers and the Central Limit Theorem). In general, males tend to possess larger rolling FSTT means than females; however, this difference is small (< 1 mm) and is unadjusted for body size (i.e., males are on average larger than females so whether or not males are in fact larger once general body size factors are considered is an open question, see [122] for more details). Median FSTT landmarks have been measured more frequently than bilateral landmarks, awarding the former larger sample sizes (Figs. 1 and 2 and Table 3).

Line plots of weighted rolling means for three bilateral landmarks (mid-supraorbital (mso–msoʹ), mid-infraorbital (mio–mioʹ), and zygion (zy–zyʹ)) illustrate stabilized mean FSTT trends, with all three landmarks yielding sex specific sample sizes > 3000 individuals (Fig. 2). The most unstable FSTT values are within the cheek region, where rolling means appear to still be in modes of active change (Fig. 2). Six bilateral landmarks have yet to stabilize, with three landmarks (supra second molar (ecm^2^–sM^2^ʹ), infra second molar (ecm_2_–iM_2_ʹ), and gonion (go–goʹ)) illustrating upward trends, while the other three landmarks (supra canine (sC–sCʹ), mid-ramus (mr–mrʹ), mid-mandibular boarder (mmb–mmbʹ)) display a downward trend (Fig. 2). The sex specific sample sizes for almost all of these landmarks are < 3000 individuals. Females exhibit larger rolling FSTT means at some bilateral landmarks; however, on average, the mean sex differences are very small (< 0.5 mm). Here, it should be noted that these sex trends are based on raw data and again and have not been subject to any body size or scale adjustments as standard in other biological domains [157, 158].

### Measurement method

#### Pooled data at median landmarks

**Fig. 3:**
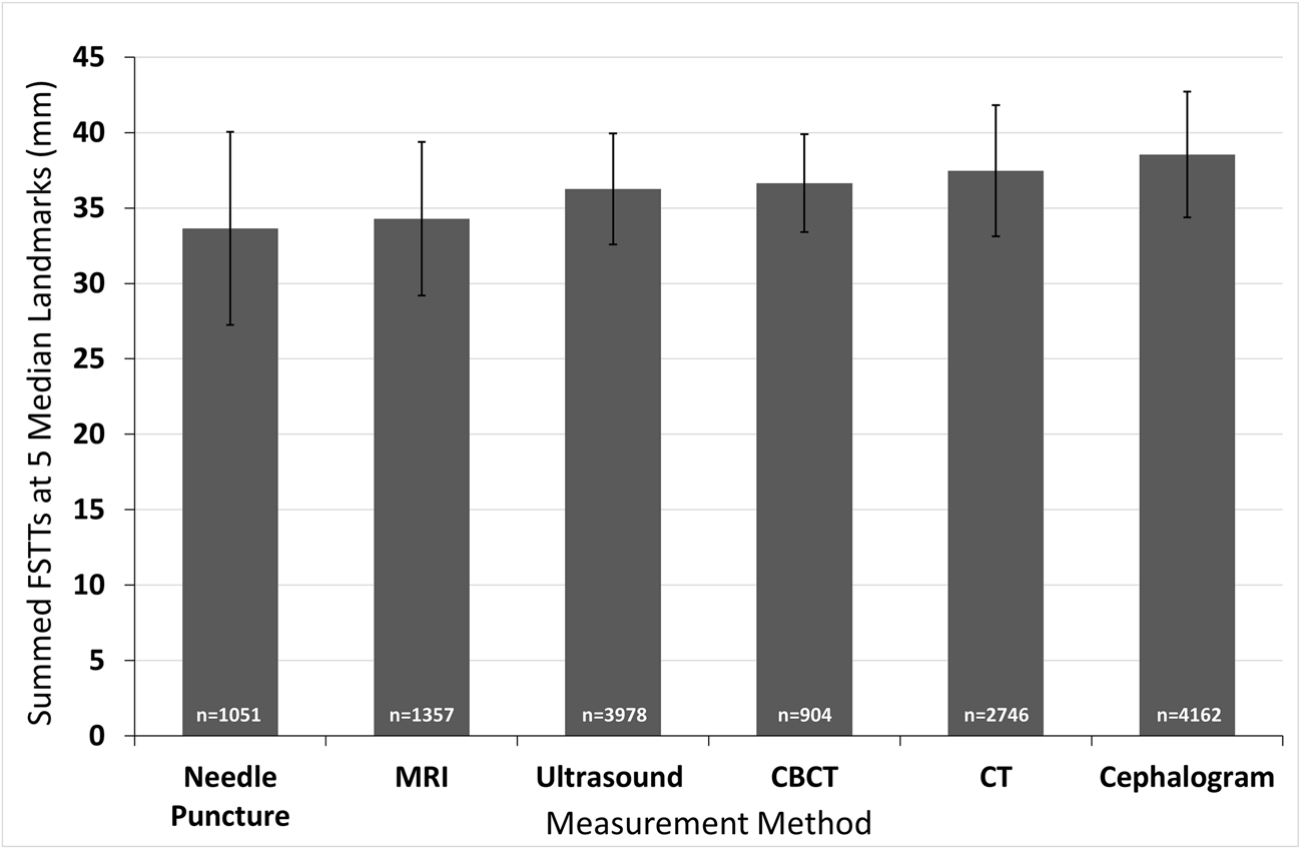
Grand means of FSTT summed across five common median landmarks. Studies contributing to these plots are presented in Supplementary Table S1. Bars represent ± 1 standard deviation of individual study means around the grand mean. The *n* values give the sample size (averaged across the five landmarks)

Pooled data for 93 studies at five common median landmarks revealed that lateral cephalograms possessed the highest mean tissue values (38.5 mm), followed by CT (37.5 mm), and then CBCT (36.6 mm) (Fig. 3). Ultrasound yielded a mid-range pooled mean (36.3 mm), while needle puncture generated the lowest pooled mean (33.6 mm). The mean tissue thickness value for MRI (34.3 mm) was closest to the needle puncture method, and slightly lower than ultrasound (Fig. 3). For almost all methods, the sample size exceeded 1000 individuals. The data with the smallest standard deviations were CBCT and ultrasound, while needle puncture yielded the greatest standard deviation (Fig. 3; Supplementary Table S1).

**Fig. 4:**
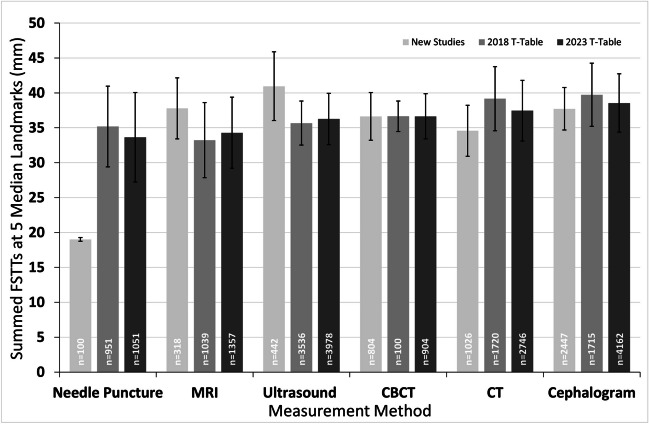
Grand means for FSTT summed across five common median landmarks by sample. Studies contributing to these plots are presented in Supplementary Table S1. Bars represent ± 1 standard deviation of individual study means around the grand mean. The *n* values give the sample size (averaged across the five landmarks)

When the analysis is broken down by sample (i.e., new data reported in this study compared to the 2018 T-Table data), the 2018 T-Table data generally follows the same trends as those reported above for the full data-suite, with the exception that MRI studies yielded the lowest mean (33.2 mm) compared to other methods (Fig. 4). However, the new data alone exhibited trends divergent from both the full data-suite and the 2018 T-Table data. For example, the new data show ultrasound yielded the highest mean tissue values (41.0 mm), followed by MRI (37.8 mm), and cephalograms (37.7 mm). Similarly, CT (34.6 mm) yielded a comparatively lower value than the 2018 T-Table data, as did needle puncture (19.0 mm) (Fig. 4; Supplementary Table S1).

#### Pooled data at bilateral landmarks

**Fig. 5:**
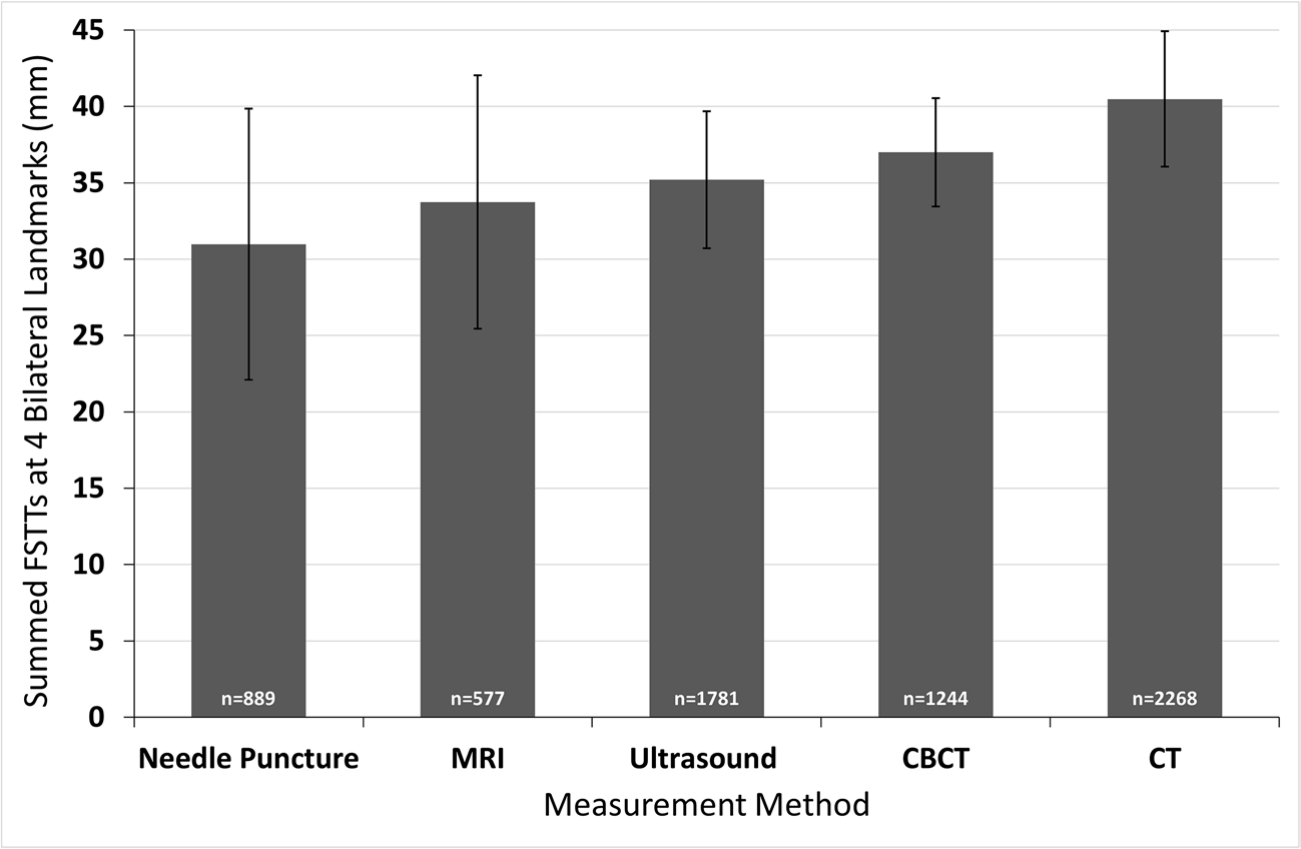
Grand means for FSTT summed across four common bilateral landmarks. Studies used to generate these plots are presented in Supplementary Table S2. Bars represent ± 1 standard deviation of individual study means around the grand mean. The *n* values give the sample size (averaged across the four landmarks)

**Fig. 6:**
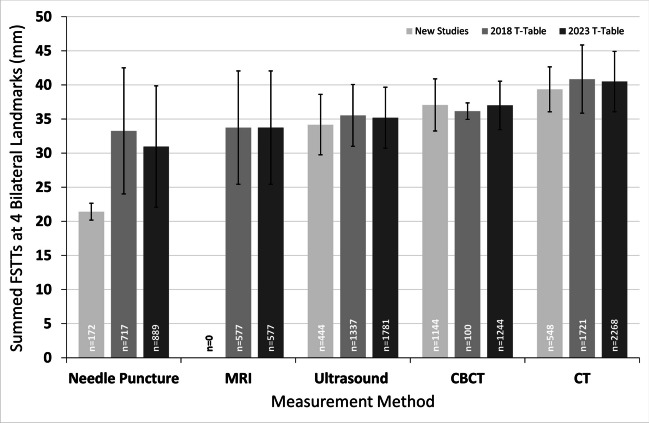
Grand means for FSTT summed across four common bilateral landmarks by sample. Studies used to generate these plots are presented in Supplementary Table S2. Bars represent ± 1 standard deviation of individual study mean around the grand mean. The *n* values give the sample size (averaged across the four landmarks)

For bilateral landmarks, the largest tissue values for the full data-suite were observed for CT (40.5 mm), followed by CBCT (37.0 mm) and ultrasound (35.2 mm), while the MRI and needle puncture studies yielded the lowest pooled FSTT means (33.7 mm and 31.0 mm, respectively) (Fig. 5). Although CT and MRI are both medical imaging methods where the subject is in supine position, CT and MRI did not yield equivalent pooled means. Despite sample sizes of the pooled data exceeding 500 individuals for both imaging modalities, the CT mean was 6.7 mm larger than the MRI pooled mean (Fig. 5; Supplementary Table S2). Similar to the median landmark data, the smallest standard deviations were observed with ultrasound and CBCT, while the largest standard deviations were observed for needle puncture and MRI (Figs. 5 and 6).

### Estimation errors of the 2023 T-Table

Performance tests of the newly generated grand means as point estimators for individuals with known FSTTs across 24 landmarks show the 2023 T-Table means produced standard errors of the estimate (*S*_est_) ranging from 1 (rhinion (rhi–rhiʹ)) to 7.7 mm (supra second molar (ecm^2^–sM^2^ʹ)), with a grand mean of 3.5 mm (Table 5). This translates to a mean absolute percentage error in the range of 16–79% (grand mean = 30%). Recalculation of the 2018 T-Table [17] standard error of the estimates with the updated v2018.1 C-Table data yielded a grand mean standard error of the estimation of 3.6 mm, indicating the updated 2023 data slightly outperform the older 2018 statistics.

**Table 5:** Estimation errors of the 2023 adult T-Table means using the v2018.1 C-Table data

	*S*_est_ (mm)	MAE (mm)	*M* (%)	*n*
Median landmarks				
op–opʹ	2.7	2.4	78	137
v–vʹ	-	-	-	0
g–gʹ	1.5	1.2	25	1264
n–seʹ	2.1	1.6	23	1311
mn–mnʹ	1.7	1.4	38	477
rhi–rhiʹ	1	0.8	41	1262
ss–snʹ	3.1	2.5	25	259
mp–mpʹ	2.8	2.1	22	1104
pr–lsʹ	3.3	2.6	25	557
id–liʹ	4.3	3.5	29	459
sm–smʹ	2.3	1.7	16	1072
pg–pgʹ	2.7	2.1	24	1080
gn–gnʹ	2.8	2.3	23	247
me–meʹ	2.9	2.1	40	1069
Bilateral landmarks				
mso–msoʹ	2.6	2	27	899
mio–mioʹ	2.9	2.1	31	882
ac–acʹ	2.6	2.1	27	390
go–goʹ	7.6	6	38	768
zy–zyʹ	3.7	3	55	709
sC–sCʹ	3.3	2.6	22	605
iC–iCʹ	2.7	2.1	18	563
ecm^2^–sM^2^ʹ	7.7	5.9	18	647
ecm_2_–iM_2_ʹ	7.5	6	23	292
mr–mrʹ	5.7	4.3	21	542
mmb–mmbʹ	5.3	4.1	28	638
Grand mean	3.5	2.8	30	-

*S*_*est*_ standard error of the estimate, *MAE* mean absolute error, *M (%) *mean percentage error, *n* sample size

## Discussion

The collection and analysis of mean facial soft tissue thickness values have been a popular pursuit to assist craniofacial identification methods. Since Welcker published the first FSTT study in 1883 [18], a total of 139 adult FSTT studies have now been published in the literature to collectively tally > 220,000 tissue thickness measurements of > 19,500 adults. In just the last 5 years, a considerable volume of new data has been added. In an effort to leverage this substantial mass of data points to triangulate upon population means, these mean data have been pooled to create the 2023 global tallied facial soft tissue depth table.

### T-Table trends: 2008–2023

Since the first adult T-Table was established in 2008 from a dataset comprising ~ 7472 individuals from 55 studies, 84 additional FSTT studies have been published and samples have almost tripled (Table 4). Every 5 years for the last 15 years, an update to the global pooled means has been provided [15–17]. A review of these pooled means reveals that the new data, contributed since 2008, have resulted in fairly minimal changes to the starting 2008 summary statistics. Since 2008, only four bilateral FSTT landmarks increased by ≥ 1.5 mm, while all other landmarks changed by ≤ 1 mm (Table 4). In general, this demonstrates that the initial 2008 T-Table data were quite informative, despite the comparatively smaller sample size.

The greatest value of the post-2008 T-Tables resides in their sequentially increasing sample size over time that allows for fluctuations in the rolling means for T-Table landmarks to be evaluated as a time series. In 2018, at just 10 years after the initial analysis, the third iteration of the T-Table did not provide enough time or data to definitively determine if pooled means had converged on population means. Just 5 years on and with substantially more data (81,790 more FSTT datapoints), the additional time window provides a much clearer view as to those patterns. Now, the convergence of rolling means on a constant unchanging value is readily apparent for median landmarks.

When the 2023 T-Table means were used as point estimators for individuals with known FSTT values (C-Table data) across 24 landmarks, the 2023 grand means outperformed the 2018 T-Table means (3.5 mm versus 3.6 mm, respectively) indicating that the updated values are superior (Table 5). While this estimation improvement is marginal (0.1 mm), meaning that for single cases in day-to-day forensic casework the difference is unlikely to be noticeable, in the long run and as applied to many cases, the improved performance of the 2023 T-Table means takes on greater meaning.

The median landmark rolling mean plots demonstrate that a sample size of at least 2500 individuals is generally required to achieve reliably stable pooled FSTT values (Fig. 1). A sample of this size typically requires 30–35 FSTT studies to be combined—thereby highlighting data reliability issues of single small, sampled studies. Some bilateral landmarks have also stabilized, but at sample sizes closer to 3000 individuals (Fig. 2), which is slightly higher than their median landmark counterparts that possess smaller standard deviations (see glabella (g–g′) or rhinion (rhi–rhi′) versus zygion (zy–zy′) or gonion (go–go′), Table 3). The most unstable bilateral FSTT values tend to be found in the cheek region, with six of these landmarks so far still failing to converge on a constant value (Fig. 2). Several factors may be contributing to these trends. These landmarks tend to be some of the largest of the face, and so possess the greatest range between individuals, particularly because they a comprised in part by fatty deposits. In comparison to median landmarks, bilateral landmarks may be more difficult to measure resulting in higher measurement error (or ‘noise’), thereby requiring larger samples to facilitate constant mean values. The continuing upward trend for some landmarks may also be a manifestation of real-world change, for instance, this may be driven by a secular trend such that more contemporary individuals hold larger bilateral FSTTs. It is additionally possible that these upwards trends could be driven by investigator preferences for a particular FSTT measurement method that yields higher values compared to other techniques. For example, both CT and CBCT have gained recent popularity for the measure of bilateral FSTTs (see below and Table 2), however, they appear to yield larger grand mean values compared to other methods (Fig. 5). Only future studies can clarify the underlying root cause for these observed trends.

### Measurement method impact

Pooling data by measurement method revealed that the methods do not appear to yield equivalent FSTT values (Figs. 3 and 5). Generally, lateral cephalograms provide the largest values for median landmarks, which is in line with prior observations [15] (Fig. 3). This is likely explained by the X-ray acquisition procedure, which involves adjustments for magnification effects and upright subject positioning [29]. In other methods, such as ultrasound, direct contact of equipment with the soft tissues risks tissue compression that may subsequently yield smaller FSTT measurements [159]. Additionally, supine body position can create thinning down the midline due to soft tissue drape and the weight of the more laterally displaced tissues, thereby yielding thicker soft tissues laterally under these effects of gravity [160–162]. As CT yielded the largest values for bilateral landmarks, this may be driven, in part, by the supine subject positioning (Fig. 5). Another important consideration for CT is the resolution provided by the slice thicknesses. When larger slice thicknesses are employed, the CT images possess a better signal-to-noise ratio but poorer resolution, which may decrease measurement accuracy [75, 163]. In the T-Table sample drawn from the literature, slice thicknesses for CT studies were highly varied, ranging from 0.5 to 7 mm. In the future, it would be useful to know exactly how slice thickness settings impact FSTT measurements and what slice thickness settings should be preferred for improved data reliability.

It is interesting to note that despite both CT and MRI being supine non-contact scan methods, MRI consistently yielded smaller FSTT values than CT in the cross-sectional context of this study (Fig. 5). These findings, first observed in [29], may suggest that the technical differences between the imaging techniques produce greater measurement effects than the common supine body positioning. Some evidence may be found for this in a study by Campenelli et al. [164], which compared 3D models of bone generated from segmented MRI and CT data. They found that CT models tended to overestimate bone size compared to 3D laser scans, while MRI models tended to underestimate the bone morphology. Similar findings have also been reported by Rathnayaka et al. [165].

### Future work

As stable pooled FSTT means for commonly measured median landmarks have been attained, the most value will be added by new studies that increase samples for bilateral landmarks, so that they too can converge upon stable rolling means.

To reduce the amount of statistical noise in the overarching FSTT dataset, it is worthwhile considering if tighter measurements can be obtained in the future and if these studies should be weighted more heavily during the averaging procedure since they are more trustworthy. This could be obtained through better sampling methods (i.e., attainment of truly random and representative samples) and/or tighter measurement protocols. As previously noted, each FSTT measurement method appears to yield slightly different FSTT values (Figs. 3 and 5), so deciding which method should provide the underlying ground truth standard is a difficult matter (especially since validation checks by direct observations on living subjects are not possible). Setting quality control standards for each data collection method (such as maximum slice thicknesses acceptable for CT data acquisition as mentioned above), though not a comprehensive solution, would be useful. While these standards could be subjectively established by a working committee, they would be better set by quantitative data that show where data accuracies breakdown under certain conditions, and under what conditions reliable data are observed. An easier and less controversial approach to reducing the data noise is simply for investigators to use better data selection strategies that produce representative samples and ensure their sample sizes are sufficiently large to test their hypotheses of interest. As statistical noise cannot ever be entirely eliminated, there is likely to be an ongoing role for data pooling and the T-Tables in the future (Figs. 4 and 6).

To maximize data utility, new FSTT studies should aim to include a base suite of common landmarks that adhere to standardized description and placement [155]. In this regard, the T-Table landmarks form a good minimum set for future investigations since these landmarks have previously been used by many investigators. Investigators can add entirely new landmarks to their studies; however, a standard set should be measured as a basis. Additionally, there is substantial value in the encouraged deposit of raw data into publicly accessible FSTT databases. This can be accomplished, for example, by contributing raw data to the Collaborative Facial Soft Tissue Depth Data Store (C-Table). Such data repositories hold the critical capacity that they can be used for validation testing newly formulated estimation models, these tests can be repeated by other investigators using the same data, and these tests can be conducted at any time since the data repository is free and open access. Currently, the critical step of validation testing newly produced FSTT estimators is rarely undertaken in FSTT studies reporting new estimators [27, 29]. For newly derived FSTT means to offer advances worthy of publication, the standard errors of the estimate must be determined and should be smaller than that of other estimators already published in the scientific literature to add value. Ideally, the validation tests should be conducted on new data not used to derive (or train) the estimators, i.e., they should concern out-of-group tests [27].

An important observation that has previously been made in the literature is the covariation of FSTTs with body mass index (BMI) [88, 122, 129, 138, 166]. This relationship will be important to award increased future attention since the mass component enables relative adjustment of FSTTs with body scale—a standard undertaking in other biological domains [157, 158], but one yet to be realized in the craniofacial identification domain [122]. Rather than treating BMI as a categorical variable for analysis, the body mass should be separately used in its native continuous data format, so that the size of its correlations with FSTTs can be appreciated in detail [122]. All future FSTT research should thereby measure the body mass of each subject in the sample, so that these relationships can be explored. Rather than reducing the mass factor to BMI (kg/m^2^), body mass should be considered in its native units (kg) as these units hold the stronger correlations with FSTTs [122].

## Conclusions

New data corresponding to > 7700 adults have been used to update pooled means and produce the 2023 version of the Global T-Table (total *N* > 19,500 adults). Rolling means show that the 2023 grand means have converged on stable values at median landmarks, while bilateral landmarks would benefit from continued data collection. Cross-sectional analysis by measurement method indicates that lateral cephalograms and CT provide large FSTTs, while needle puncture provides the smallest values. Within-group validation tests of these updated 2023 Global T-Table values show these means provide slightly more accurate FSTT estimates than the 2018 T-Table data. To maximize the quality and utility of FSTT data, future research should devise optimal data collection strategies that produce less noisy datasets (i.e., reduce measurement and sampling errors) and use the T-Table landmarks as a minimum landmark suite for additional data collection.

### Supplementary Information

Below is the link to the electronic supplementary material.

Supplementary file1 (DOCX 147 KB)

## Data Availability

All study-specific data used in this investigation are available in the scientific domain and can be readily accessed in the published scientific literature per citations provided in Table 1.

## Declarations

### Disclaimer

The views and opinions expressed herein are entirely those of the authors. They are not to be construed as official views of any institutions, editorial boards, or governing boards to which the authors may be affiliated.

## References

[1] Helmer R, Koschorek F, Terwey B, Frauen T (1986). Dickenmessung der Gesichtsweichteile mit Hilfe der Kernspin-Tomographie zum Zwecke der Identifizierung. Archives für Kriminologie.

[2] Prag J, Neave R (1997). Making faces: using forensic and archaeological evidence.

[3] Gerasimov MM (1955) Vosstanovlenie lica po cerepu. Izdat. Akademii Nauk SSSR Moskva

[4] Gerasimov MM (1949) Osnovy Vostanovleniia Litsa po Cherepo. Izdat. Akademii Nauk SSSR Moskva

[5] Ullrich H, Stephan CN (2016). Mikhail Mikhaylovich Gerasimov’s authentic approach to plastic facial reconstruction. Anthropologie (Brno).

[6] Stephan C (2015). Facial approximation – from facial reconstruction synonym to face prediction paradigm. J Forensic Sci.

[7] Stephan CN (2018) A workshop on Gerasimov’s plastic facial reconstruction—17th meeting of the International Association of Craniofacial Identification, Brisbane, July, 2017. Anthropologie (Brno) 56: 68–71. 10.26720/anthro.18.02.12.1

[8] Simpson E, Henneberg M (2002). Variation in soft-tissue thicknesses on the human face and their relation to craniometric dimensions. Am J Phys Anthropol.

[9] Stephan CN, Sievwright E (2018). Facial soft tissue thickness (FSTT) estimation models—and the strength of correlations between craniometric dimensions and FSTTs. Forensic Sci Int.

[10] Wilkinson C (2004). Forensic facial reconstruction.

[11] Ullrich H (1958). Die methodischen Grundlagen des plastischen Rekonstruktionsverfahrens nach Gerasimov. Zeitschrift fur Morpholologie und Anthropologie.

[12] Ullrich H (1966) Kritische Bemerkungen zur plastischen Rekonstruktionsmethode nach Gerasimov auf Grund personlicher Erfahrugen. Ethnographisch-archäologische Zeitschrift 7: 111–23

[13] Houlton TM, Jooste N, Steyn M (2021). Testing regression and mean model approaches to facial soft-tissue thickness estimation. Med Sci Law.

[14] Niinimäki S, Karttunen A. (2007) Study on the facial tissue thickness of the Finns. In: Buzug TM, Sigl KM, Bongartz J, Prüfer K, eds. Facial reconstruction: forensic, medical and archeological methods of the reconstruction of soft facial parts Gesichtsrekonstruktion: Forensische, Medizinische Und Archäologische Methoden Der Gesichtsweichteilrekonstruktion. Luchterhand Munich. pp 95–120

[15] Stephan CN, Simpson EK (2008). Facial soft tissue depths in craniofacial identification (part I): an analytical review of the published adult data. J Forensic Sci.

[16] Stephan C (2014). The application of the central limit theorem and the law of large numbers to facial soft tissue depths: T-Table robustness and trends since 2008. J Forensic Sci.

[17] Stephan CN (2017). 2018 Tallied facial soft tissue thicknesses: adult and sub-adult data. Forensic Sci Int.

[18] Welcker H (1883). Schiller’s Schädel und Todtenmaske, nebst Mittheilungen über Schädel und Todtenmaske Kant’s.

[19] His W (1895) Anatomische Forschungen über Johann Sebastian Bach’s Gebeine und Antlitz nebst Bemerkungen über dessen Bilder. Abhandlungen der mathematisch-physikalischen Classe der Königlichen Sächsischen Gesellschaft der Wissenschaften 22: 379–420

[20] Welcker H (1896). Das Profil des menschlichen Schädels mit Röntgenstrahlen am Lebenden dargestellt. Korrespondenz-Blatt der Deutschen Gesellschaft für Anthropologie Ethnologie und Urgeschichte.

[21] De Greef S, Claes P, Vandermeulen D, Mollemans W, Suetens P, Willems G (2006). Large-scale *in-vivo* Caucasian soft tissue thickness database for craniofacial reconstruction. Forensic Sci Int.

[22] Phillips VM, Smuts NA (1996). Facial reconstruction: utilization of computerized tomography to measure facial tissue thickness in a mixed racial population. Forensic Sci Int.

[23] Hwang H-S, Park M-K, Lee W-J, Cho J-H, Kim B-K, Wilkinson CM (2012). Facial soft tissue thickness database for craniofacial reconstruction in Korean adults. J Forensic Sci.

[24] Sahni D, Jit I, Gupta M, Singh P, Suri S (2002) Preliminary study on facial soft tissue thickness by magnetic resonance imaging in Northwest Indians. Forensic Science Communications. https://archives.fbi.gov/archives/about-us/lab/forensic-science-communications/fsc/jan2002/sahni.htm. Accessed 1 January 2023

[25] Sahni D, Sanjeev SD, Jit I, Singh P (2008). Facial soft tissue thickness in northwest Indian adults. Forensic Sci Int.

[26] Stephan CN, Simpson EK (2008). Facial soft tissue depths in craniofacial identification (part II): an analytical review of the published sub-adult data. J Forensic Sci.

[27] Stephan CN (2015). Accuracies of facial soft tissue depth means for estimating ground truth skin surfaces in forensic craniofacial identification. Int J Legal Med.

[28] Stephan CN, Munn L, Caple J (2015). Facial soft tissue thicknesses: noise, signal and P. Forensic Sci Int.

[29] Stephan CN, Meikle B, Freudenstein N, Taylor R, Claes P (2019). Facial soft tissue thicknesses in craniofacial identification: data collection protocols and associated measurement errors. Forensic Sci Int.

[30] Moore DS, McCabe GP (2003). Introduction to the practice of statistics.

[31] Norman GR, Streiner DL (2000). Biostatistics: the bare essentials.

[32] Kollmann J, Büchly W (1898). Die Persistenz der Rassen und die Reconstruction der Physiognomie prähistorischer Schädel. Archiv für Anthropologie.

[33] Birkner F (1904). Beiträge zur Rassenanatomie der Gesichtsweichteile. Corr Bl Anthrop Ges Jhg.

[34] Fischer E (1905). Anatomische Untersuchungen an den Kopfweichteilen zweier Papua. CorrBLAnthrop Ges Jhg.

[35] Czekanowski J (1907). Untersuchungen über das Verhältnis der Kopfmaße zu den Schädelmaßen. Archiv für Anthropologie.

[36] Eggeling HV (1909) Anatomische Untersuchungen an den Köpfen von ver Hereros, einem Herero- und einem Hottentottenkind. In: Schultze L, ed. Forschungsreise im westlichen und zentralen Südafrika. Denkschriften Jena. pp 323–48

[37] Stadtmüller F (1922). Zur Beurteilung der plastischen Rekonstruktionsmethode der Physiognomie auf dem Schädel. Zeitschrift für Morpholologie und Anthropologie.

[38] Burkitt AN, Lightoller GHS (1923). Preliminary observations on the nose of the Australian aboriginal with a table of aboriginal head measurements. J Anat.

[39] Edelman H (1938). Die Profilanalyse: Eine Studie an photographischen und röntgenographischen Durchdringungsbildern. Zeitschrift fur Morpholologie und Anthropologie.

[40] Suzuki H (1948). On the thickness of the soft parts of the Japanese face. J Anthropol Soc Nippon.

[41] Stewart TD (1954) Evaluation of evidence from the skeleton. In: Gradwohl RBH, ed. Leg Med. C. V. Mosby St. Louis. pp 407–50

[42] Subtelny JD (1959). A longitudinal study of soft tissue facial structures and their profile characteristics, defined in relation to underlying skeletal structures. Am J Orthod.

[43] Ogawa H (1960). Anatomical study on the Japanese head by X-ray cephalometry. J Tokyo Dental Coll Soc [Shika Gakuho].

[44] Leopold D (1968). Identifikation durch Schädeluntersuchung unter besonderer Berücksichtigung der Superprojektion.

[45] Helwin H (1969). Die Profilanalyse, eine Möglichkeit der Identifizierung unbekannter Schädel. Gegenbaurs Morphol Jahrb.

[46] Sutton PRN (1969). Bizygomatic diameter: the thickness of the soft tissues over the zygions. Am J Phys Anthropol.

[47] Rhine JS, Campbell HR (1980). Thickness of facial tissues in American blacks. J Forensic Sci.

[48] Sarnäs K-V, Solow B (1980). Early adult changes in the skeletal and soft-tissue profile. Eur J Orthod.

[49] Rhine S (1983). Tissue thickness for Southwestern Indians.

[50] Rhine JS, Moore CE (1984) Tables of facial tissue thickness of American Caucasoids in forensic anthropology. Maxwell Museum Technical Series 1

[51] Forrest AS (1985). An investigation into the relationship between facial soft tissue thickness and age in Australian Caucasion cadavers.

[52] Dumont ER (1986). Mid-facial tissue depths of white children: an aid in facial feature reconstruction. J Forensic Sci.

[53] Lebedinskaya GV, Veselovskaya EV (1986) Ultrasonic measurements of the thickness of soft facial tissue among the Bashkirs. Annales Academiae Scientiarium Fennicae SerA 5 Medica 175: 91–5 3548496

[54] George RM (1987). The lateral craniographic method of facial reconstruction. J Forensic Sci.

[55] Nanda RS, Meng H, Kapila S, Goorhuis J (1990) Growth changes in the soft tissue facial profile. Angle Orthod 60: 177–90. 10.52010/ijom.1991.17.1.4 10.1043/0003-3219(1990)060<0177:GCITST>2.0.CO;2 2389850

[56] O’Grady JF, Taylor RG, Clement JG (1990). Facial tissue thickness: a study of cadavers in Melbourne.

[57] Ligthelm-Bakker ASWMR, Prahl-Andersen B, Wattel E, Uljee IH (1991). A new method for locating anterior skeletal landmarks from soft tissue measurements. J Biol Buccale.

[58] Lebedinskaya GV, Balueva TS, Veselovskaya EV.(1993) Principles of facial reconstruction. In: İşcan MY, Helmer RP, eds. Forensic analysis of the skull. Wiley-Liss New York. pp 183–98

[59] Formby WA, Nanda RS, Currier GF (1994). Longitudinal changes in the adult facial profile. Am J Orthod Dentofacial Orthop.

[60] Michelow BJ, Guyuron B (1995). The chin: skeletal and soft-tissue components. Plast Recon Surg.

[61] Anderson W (1996). The correlation between soft tissue thickness and bony proportions of the skull and how they relate to facial reconstruction.

[62] Aulsebrook WA, Becker PJ, İşcan MY (1996). Facial soft-tissue thickness in the adult male Zulu. Forensic Sci Int.

[63] Blythe T (1996). A re-assessment of the Rhine and Moore Technique in forensic facial reconstruction.

[64] Kasai K (1998). Soft tissue adaptability to hard tissues in facial profile. Am J Dentofacial Orthop.

[65] Miyasaka S (1999). Progress in facial reconstruction technology. Forensic Sci Rev.

[66] Garlie TN, Saunders SR (1999). Midline facial tissue thicknesses of subadults from a longitudinal radiographic study. J Forensic Sci.

[67] Manhein MH, Listi GA, Barsley RE, Musselman R, Barrow NE, Ubelaker DH (2000). In vivo facial tissue depth measurements for children and adults. J Forensic Sci.

[68] El-Mehallawi IH, Soliman EM (2001). Ultrasonic assessment of facial soft tissue thickness in adult Egyptians. Forensic Sci Int.

[69] Smith SL, Buschang PH (2001). Midsagittal facial tissue thickness of children and adolescents from the Montreal growth study. J Forensic Sci.

[70] Sutisno M (2003). Human facial soft-tissue thickness and its value in forensic facial reconstruction.

[71] Domaracki M, Stephan CN (2006). Facial soft tissue thicknesses in Australian adult cadavers. J Forensic Sci.

[72] Niinimaki S, Karttunen A (2006) Finnish facial tissue thickness study. In: Herva V-P, ed. Proceedings of the 22nd Nordic Archaeological Conference. Gummerus Kirjapaino Oy University of Oulu. pp 343–52

[73] Martin R, Saller K (1957). Lehrbuch der Anthropologie.

[74] Helmer R (1984). Schädelidentifizierung durch elekroniesche Bildmischung: Zugleich ein Beitrag zur Konstitutionsbiometrie und Dickenmessung der Gesichtsweichteile.

[75] Montagu A (1935). The location of the nasion in the living. Am J Phys Anthropol.

[76] Kim K-D, Ruprecht A, Wang G, Lee JB, Dawson DV, Vannier MW (2005). Accuracy of facial soft tissue thickness measurements in personal computer-based multiplanar reconstructed computed tomographic images. Forensic Sci Int.

[77] Smith SL, Throckmorton GS (2006). Comparability of radiographic and 3D-ultrasound measurements of facial midline tissue depths. J Forensic Sci.

[78] Vander Pluym J, Shan WW, Taher Z (2007). Use of magnetic resonance imaging to measure facial soft tissue depth. Cleft Palate Craniofac J.

[79] Suazo GIC, Cantín LM, Zavando MDA, Perez RFJ, Torres MSR (2008). Comparisons in soft-tissue thicknesses on the human face in fresh and embalmed corpses using needle puncture method. Int J Morphol.

[80] Codinha S (2009) Facial soft tissue thicknesses for the Portuguese adult population. Forensic Sci Int 184: 80.e1-.e7. 10.1016/j.forsciint.2008.11.011 10.1016/j.forsciint.2008.11.011 19124207

[81] Tedeschi-Oliveira SV, Melani RFH, de Almeida N, de Paiva LA (2009). Facial soft tissue thickness of Braziallian adults. Forensic Sci Int.

[82] Tilotta F, Richard F, Glaunes J (2009). Construction and analysis of a head CT-scan database for craniofacial reconstruction. Forensic Sci Int.

[83] Utsuno H, Kageyama T, Uchida K, Yoshino M, Oohigashi S, Miyazawa H (2010) Pilot study of facial soft tissue thickness differences among three skeletal classes in Japanese females. Forensic Sci Int 195: 165.e1-.e5. 10.1016/j.forsciint.2009.10.013 10.1016/j.forsciint.2009.10.013 19942386

[84] Cavanagh D, Steyn M (2011) Facial reconstruction: soft tissue thickness values for South African black females. Forensic Sci Int 206: 215.e1-.e7. 10.1016/j.forsciint.2011.01.009 10.1016/j.forsciint.2011.01.009 21288672

[85] Chan WN, Listi GA, Manhein MH (2011). *In vivo* facial tissue depth study of Chinese-American adults in New York City. J Forensic Sci.

[86] Chen F, Chen Y, Yu Y, Qiang Y, Liu M, Fulton D (2011) Age and sex related measurement of craniofacial soft tissue thickness and nasal profile in the Chinese population. Forensic Sci Int 212: 272.e1-.e6. 10.1016/j.forsciint.2011.05.027 10.1016/j.forsciint.2011.05.027 21715112

[87] Kurkcuoglu A, Pelin C, Ozener B, Zagyapan R, Sahinoglu Z, Yazici AC (2011). Facial soft tissue thickness in individuals with different occlusion patterns in adult Turkish subjects. Homo.

[88] Dong Y, Huang L, Feng Z, Bai S, Wu G, Zhao Y (2012) Influence of sex and body mass index on facial soft tissue thickness measurements of the northern Chinese adult population. Forensic Sci Int 222: 396.e1-.e7. 10.1016/j.forsciint.2012.06.004 10.1016/j.forsciint.2012.06.004 22738738

[89] Paneková P, Beňuš R, Masnicová S, Obertová Z, Grunt J (2012) Facial soft tissue thicknesses of the mid-face for Slovak population. Forensic Sci Int 220: 293.e1-.e6. 10.1016/j.forsciint.2012.02.015 10.1016/j.forsciint.2012.02.015 22430009

[90] Sipahioglu S, Ulubay H, Diren HB (2012) Midline facial soft tissue thickness database of Turkish population: MRI study. Forensic Sci Int 219: 282.e1-.e38. 10.1016/j.forsciint.2011.11.017 10.1016/j.forsciint.2011.11.017 22154437

[91] Guyomarc'h P, Santos F, Dutailly B, Coqueugniot H (2013) Facial soft tissue depths in French adults: variability, specificity and estimation. Forensic Sci Int 231: 411.e1-.e10. 10.1016/j.forsciint.2013.04.007 10.1016/j.forsciint.2013.04.007 23684263

[92] Saxena T, Panat SR, Sangamesh NC, Choudhary A, Aggarwal A, Yadav N (2012). Facial soft tissue thickness in North Indian adult population. J Indian Acad Oral Med Radiol.

[93] de Almeida NH, Michel-Crosato E, de Paiva LA, Biazevic MG (2013). Facial soft tissue thickness in the Brazilian population: new reference data and anatomical landmarks. Forensic Sci Int.

[94] Perlaza Ruiz N (2013) Facial soft tissue thickness of Colombian adults. Forensic Sci Int 229: 160.e1-.e9. 10.1016/j.forsciint.2013.03.017 10.1016/j.forsciint.2013.03.017 23587676

[95] Bulut O, Sipahioglu S, Hekimoglu B (2014). Facial soft tissue thickness database for craniofacial reconstruction in the Turkish adult population. Forensic Sci Int.

[96] Gorbenko I, Mikołajczyk K, Iarovyi I, Kubik T, Kałużyński K. (2014) A new method of automatic craniometric landmarks definition and soft tissue thickness measurement based on MRI data. In: Piętka E, Kawa J, Wieclawek W, eds. Information technologies in biomedicine. Springer Switzerland. pp 115–26

[97] Parks CL, Richard AH, Monson KL (2014) Preliminary assessment of facial soft tissue thickness utilizing three-dimensional computed tomography models of living individuals. Forensic Sci Int 237: 146.e1-.e10. 10.1016/j.forsciint.2013.12.043 10.1016/j.forsciint.2013.12.043 24529417

[98] Baillie LJ, Ali Mirijali S, Niven BE, Blyth P, Dias GJ (2015). Ancestry and BMI influences on facial soft tissue depths for a cohort of Chinese and Caucasoid women in Dunedin, New Zealand. J Forensic Sci.

[99] Chung JH, Hsu HT, Chen HT, Huang GS, Shaw KP (2015) A CT-scan database for the facial soft tissue thickness of Taiwan adults. Forensic Sci Int 253: 132.e1-.e11. 10.1016/j.forsciint.2015.04.028 10.1016/j.forsciint.2015.04.028 26028278

[100] Drgáčová A, Dupej J, Velemínská J (2016) Facial soft tissue thicknesses in the present Czech population. Forensic Sci Int 260: 106.e1-.e17. 10.1016/j.forsciint.2016.01.011 10.1016/j.forsciint.2016.01.011 26860069

[101] Jia L, Qi B, Yang J, Zhang W, Lu Y, Zhang H-L (2016) Ultrasonic measurement of facial tissue depth in a Northern Chinese Han population. Forensic Sci Int 259: 247.e1-.e6. 10.1016/j.forsciint.2015.12.012 10.1016/j.forsciint.2015.12.012 26778588

[102] Wang J, Zhao X, Mi C, Raza I (2016) The study on facial soft tissue thickness using Han population in Xinjiang. Forensic Sci Int 266: 585.e1-.e5. 10.1016/j.forsciint.2016.04.032 10.1016/j.forsciint.2016.04.032 27216250

[103] Thiemann N, Keil V, Roy U (2017). In vivo facial soft tissue depths of a modern adult population from Germany. Int J Legal Med.

[104] Kang SG, Lee YJ, Park YG (2003). A comparative study of soft tissue profile between Korean and Caucasian young adults under NHP. Korean J Orthod.

[105] Bellmann D, Fuchs T, Weidenbusch A et al. (2007) Computer-aided measurement of the tissue thickness of deceased persons with computer tomography scans of the head. In: Buzug TM, Sigl KM, Bongartz J, Prüfer K, eds. Facial reconstruction: forensic, medical and archeological methods of the reconstruction of soft facial parts Gesichtsrekonstruktion: Forensische, Medizinische Und Archäologische Methoden Der Gesichtsweichteilrekonstruktion. Luchterhand Munich. pp. 21–39

[106] Shimofusa R, Yamamoto S, Horikoshi T, Yokota H, Iwase H (2009). Applicability of facial soft tissue thickness measurements in 3-dimensionally reconstructed multidetector-row CT images for forensic anthropological examination. Leg Med.

[107] Salazar CB, Matamala DZ, Cantín M, Galdames IS (2010). Facial tissue thickness in Chilean cadavers with medico-legal purposes. Int J Odontostomatol.

[108] Torres Muñoz SR, Cantín M, Pérez Rojas FJ, Suazo Galdames I (2011). Evaluation of facial asymmetry using soft-tissue thickness for forensic purposes. Int J Morphol.

[109] Kamak H, Celikoglu M (2012). Facial soft tissue thickness among skeletal malocclusions: is there a difference?. Korean J Orthod.

[110] Cha K (2013). Soft-tissue thickness of South Korean adults with normal facial profiles. Korean J Orthod.

[111] MacNeil JAB, Peckmann TR, Mussett M (2013). Asymmetry in forensic three-dimensional facial reconstruction: an assessment of facial asymmetry in adult First Nations Nova Scotian facial soft tissue depth data. Can Soc Forensic Sci J.

[112] Utsuno H, Kageyama T, Keiichi U, Kibayashi K (2014). Facial soft tissue thickness differences among three skeletal classes in Japanese population. Forensic Sci Int.

[113] Celikoglu M, Buyuk SK, Ekizer A, Sekerci AE, Sisman Y (2015). Assessment of the soft tissue thickness at the lower anterior face in adult patients with different skeletal vertical patterns using cone-beam computed tomography. Angle Orthod.

[114] Gungor K, Bulut O, Hizliol I, Hekimoglu B, Gurcan S (2015). Variations of midline facial soft tissue thicknesses among three skeletal classes in Central Anatolian adults. Leg Med.

[115] Jeelani W, Fida M, Shaikh A (2015). Facial soft tissue thickness among three skeletal classes in adult Pakistani subjects. J Forensic Sci.

[116] Jeelani W, Fida M, Shaikh A (2015) Facial soft tissue thickness among various vertical facial patterns in adult Pakistani subjects. Forensic Sci Int 257: 517.e1-.e6. 10.1016/j.forsciint.2015.09.006 10.1016/j.forsciint.2015.09.006 26476716

[117] Peckmann TR, Harris M, Huculak M, Pringle A, Fournier M (2015). In vivo facial tissue depth for Canadian Mi’kmaq adults: a case study from Nova Scotia, Canada. J Forensic Leg Med.

[118] Hamid S, Abuaffan AH (2016). Facial soft tissue thickness in a sample of Sudanese adults with different occlusions. Forensic Sci Int.

[119] Jazmati HM, Ajaj MA, Hajeer MY (2016). Assessment of facial soft tissue dimensions in adult patients with different sagittal skeletal classes using cone beam computed tomography. J Contemp Dent Pract.

[120] Kotrashetti VS, Mallapur MD (2016). Radiographic assessment of facial soft tissue thickness in South Indian population - an anthropologic study. J Forensic Leg Med.

[121] Lodha A, Mehta M, Patel M, Menon SK (2016). Facial soft tissue thickness database of Gujarati population for forensic craniofacial reconstruction. Egypt J Forensic Sci.

[122] Stephan CN, Priesler R, Bulut O, Bennett MB (2016). Turning the tables of sex distinction in craniofacial identification: why females possess thicker facial soft tissues than males, not vice versa. Am J Phys Anthropol.

[123] Kaur K, Sehrawat JS, Bahadur R (2017). Sex dependent variations in craniofacial soft-tissue thicknesses estimated from MRI and CT scans: a pilot study based on Northwest Indian subjects. Int J Diagn Imaging.

[124] Kimura Y, Okazaki K (2018). Facial soft tissue depth measured using ultrasonography: towards facial approximation for Japanese crania. Anthropol Sci (Japanese Series).

[125] Perović T, Blažej Z (2018) Male and female characteristics of facial soft tissue thickness in different orthodontic malocclusions evaluated by cephalometric radiography. Med Sci Monit 24: 3415–24. 10.12659/MSM.907485 10.12659/MSM.907485 PMC5994140 29791323

[126] Sandamini H, Jayawardena A, Batuwitage L et al (2018) Facial soft tissue thickness trends for selected age groups of Sri Lankan adult population. Forensic Sci Int 293: 102.e1-.e11. 10.1016/j.forsciint.2018.10.001 10.1016/j.forsciint.2018.10.001 30391103

[127] Sattar A, Ahmed I, Khan T (2018). Assessment of the soft tissue chins thickness with different skeletal vertical patterns in Pakistani adults. J Dent Hyg.

[128] Stephan CN, Preisler R (2018) *In vivo* facial soft tissue thicknesses of adult Australians. Forensic Sci Int 282: 220.e1-.e12. 10.1016/j.forsciint.2017.11.014 10.1016/j.forsciint.2017.11.014 29198591

[129] Toneva D, Nikolova S, Georgiev I (2018). Facial soft tissue thicknesses in Bulgarian adults: relation to sex, body mass index and bilateral asymmetry. Folia Morphol.

[130] Ayoub F, Saadeh M, Rouhana G, Haddad R (2019) Midsagittal facial soft tissue thickness norms in an adult mediterranean population. Forensic Sci Int 294: 217.e1-.e7. 10.1016/j.forsciint.2018.10.021 10.1016/j.forsciint.2018.10.021 30455033

[131] De Donno A, Sablone S, Lauretti C (2019). Facial approximation: soft tissue thickness values for Caucasian males using cone beam computer tomography. Leg Med.

[132] Meundi MA, David CM (2019). Application of cone beam computed tomography in facial soft tissue thickness measurements for craniofacial reconstruction. J Oral Maxillofac Pathol.

[133] Meundi MA, David CM (2019) Morphometric analysis of facial soft tissue thickness for sexual dimorphism: a cone beam computed tomography study. Int J Forensic Med Toxicol Sci 4: 60–7. 10.18231/j.ijfmts.2019.014

[134] Meundi MA, David CM (2019). Facial soft tissue thickness in South Indian adults with varied occlusions–a cone beam computed tomography study. J Indian Acad Oral Med Radiol.

[135] Somos CP, Rea PM, Shankland S, Kranioti EF. (2019) Medical imaging and facial soft tissue thickness studies for forensic craniofacial approximation: a pilot study on modern cretans. In: Rea P, ed. Biomedical visualisation. Springer Cham. pp. 71–86. 10.1007/978-3-030-14227-8_6 31313259

[136] Chu G, Han M, Ji L (2020). Will different sagittal and vertical skeletal types relate the soft tissue thickness: a study in Chinese female adults. Leg Med.

[137] Deng C, Wang D, Chen J (2020). Facial soft tissue thickness in Yangtze River delta Han population: accurate assessment and comparative analysis utilizing Cone-Beam CT. Leg Med.

[138] Eftekhari-Moghadam AR, Latifi SM, Nazifi HR, Rezaian J (2020). Influence of sex and body mass index on facial soft tissue thickness measurements in an adult population of southwest of Iran. Surg Radiol Anat.

[139] Imanimoghaddam M, Ketabchi G, Tohidi E, Ardakani AH (2020) Thickness of facial soft tissue in adult patients with class I, II and III skeletal patterns in digital lateral cephalometery. J Dent Mater Tech 9: 116–22. 10.22038/JDMT.2020.45060.1334

[140] Kunnath JT, Subrahmanya RM, Dhillon H (2020). Assessment of facial soft tissue thickness in individuals having skeletal class II alocmclusion. World J Dent.

[141] Sarilita E, Rynn C, Mossey PA, Black S, Oscandar F (2020). Facial average soft tissue depth variation based on skeletal classes in Indonesian adult population: a retrospective lateral cephalometric study. Leg Med.

[142] Sodawala J, Akolkar A, Sodawala F, Gandhi S, Hamdani S, Ali SM (2020). Comparison of soft tissue chin thickness at different levels of chin in subjects with various growth patterns. Indian J Dent Res.

[143] Tanaka C, Utsuno H, Makino Y (2020). Facial soft tissue thickness of the Japanese population determined using post mortem computed tomography images. Forensic Imaging.

[144] Beaini TL, Miamoto P, Duailibi-Neto EF, Tedeschi-Oliveira SV, Chilvarquer I, Melani RFH (2021). Facial soft tissue depth measurements in cone-beam computed tomography: a study of a Brazilian sample. Leg Med.

[145] de Barros F, da Costa SM, Kuhnen B, Scarso Filho J, Gonçalves M, Fernandes CMS (2021). Midsagittal and bilateral facial soft tissue thickness: a cone-beam computed tomography assessment of Brazilian living adults. Forensic Imaging.

[146] Navic P, Sinthubua A, Prasitwattanaseree S, Mahakkanukrauh P (2021). Facial soft tissue thickness for a Thai population using needle puncture technique application for forensic facial reconstruction. Int Med J.

[147] Patil HS, Golwalkar S, Chougule K, Kulkarni NR (2021). Comparative evaluation of soft tissue chin thickness in adult patients with skeletal class II malocclusion with various vertical growth patterns: a cephalometric study. Folia Med.

[148] Abdulal G, Osman A, Abyad A (2022) Evaluation of facial soft-tissue morphology among different vertical skeletal profile. Eur J Orthod 18: 117–34. 10.19044/esj.2022.v18n11p117

[149] Alhumadi A, Al-Khafaji TJ, Alyassiri AMH, Alhamadi WW (2022). Gender differences in lower facial soft tissue thickness among different skeletal patterns, based on soft tissue cephalometric analysis. J Orthod Sci.

[150] Chaudhary A, Giri J, Gyawali R, Pokharel PR (2022). A retrospective study comparing nose, lip, and chin morphology in class I, class II, and class III skeletal relationships in patients visiting to the Department of Orthodontics, BPKIHS: a cephalometric study. Int J Dent.

[151] De Donno A, Mele F, Angrisani C (2022). Facial approximation for identification purposes: soft tissue thickness in a Caucasian population. Sex and age-related variations. J Forensic Odonto-Stomatol.

[152] Moritsugui DS, Fugiwara FVG, Vassallo FNS, Mazzilli LEN, Beaini TL, Melani RFH (2022). Facial soft tissue thickness in forensic facial reconstruction: impact of regional differences in Brazil. PLoS ONE.

[153] Shehata TI, Khattab N, Wahab TMA, Ekram AM, Elbashar YH (2022). Imaging analysis of cone beam computed tomography for present Egyptians facial soft tissue thicknesses. J Opt.

[154] Wang D, Zhang Q, Zeng N, Wu Y (2022). Age-related changes in facial soft tissue of Han Chinese: a computed tomographic study. Dermatol Surg.

[155] Caple J, Stephan CN (2016). A standardized nomenclature for craniofacial and facial anthropometry. Int J Legal Med.

[156] Hona TWP, Stephan C (2022). Cephalometric landmark standards and recent trends in craniofacial identification (2018–22): avoiding imposters by describing variant landmarks as supplemental. Forensic Imaging.

[157] Huxley JS (1972). Problems of relative growth.

[158] Brown JH, West GB, Enquist BJ, Brown JH, West GB (2000). Scaling in biology: patterns and processes, causes and consequences. Scaling in biology.

[159] Meikle B, Stephan CN (2020). B-mode ultrasound measurement of facial soft tissue thickness for craniofacial identification: a standardized approach. J Forensic Sci.

[160] Munn L, Stephan CN (2018). Changes in face topography from supine-to-upright position—and soft tissue correction values for craniofacial identification. Forensic Sci Int.

[161] Ozsoy U, Sekerci R, Ogut E (2015). Effect of sitting, standing, and supine body positions on facial soft tissue: detailed 3D analysis. Int J Oral Maxillofac Surg.

[162] Bulut O, Liu C-YJ, Koca F, Wilkinson C (2017). Comparison of three-dimensional facial morphology between upright and supine positions employing three-dimensional scanner from live subjects. Leg Med.

[163] Ford JM, Decker SJ (2016). Computed tomography slice thickness and its effects on three-dimensional reconstruction of anatomical structures. J Forensic Radiol Imaging.

[164] Campanelli V, Howell S, Hull M (2020). Morphological errors in 3D bone models of the distal femur and proximal tibia generated from magnetic resonance imaging and computed tomography determined using two registration methods. Comput Methods Biomech Biomed Eng Imaging Vis.

[165] Rathnayaka K, Sahama T, Schuetz MA, Schmutz B (2011). Effects of CT image segmentation methods on the accuracy of long bone 3D reconstructions. Med Eng Phys.

[166] De Greef S, Vandermeulen D, Claes P, Suetens P, Willems G (2009). The influence of sex, age and body mass index on facial soft tissue depths. J Oral Maxillofac Pathol.

