# Supplementary material for: Global facial soft tissue thicknesses for craniofacial identification (2023): a review of 140 years of data since Welcker’s first study
Source: Int J Legal Med. 2023 Oct 7;138(2):519–35. doi: 10.1007/s00414-023-03087-x (PMC10861615; doi:10.1007/s00414-023-03087-x)
Supplement: Supplementary file 1 — Supplementary file1 (DOCX 147 KB) [file 414_2023_3087_MOESM1_ESM.docx]

**Supplementary Tables**

**Article Title:** Global Facial Soft Tissue Thicknesses for Craniofacial Identification (2023): A Review of 140 years of Data since Welcker’s First Study

**DOI:** <https://doi.org/10.1007/s00414-023-03087-x>

**Journal Name:** International Journal of Legal Medicine

**Author Names:** Te Wai Pounamu T. Hona and Carl N. Stephan

**Affiliations:** Laboratory for Human Craniofacial and Skeletal Identification (HuCS-ID Lab), School of Biomedical Sciences, The University of Queensland, Brisbane, 4072, Australia

**Corresponding Author Email:** [t.hona@uq.net.au](mailto:t.hona@uq.net.au)

**Table S1** Studies and sample sizes used to generate Fig. 3 & Fig. 4

|  | **Measurement Method** | **No. Studies** | **Pooled n** | **References** |
| --- | --- | --- | --- | --- |
| 2023 T-Table | Needle Puncture | 24 | 1051 | [1-22]; Fisher and Moorman (year unknown) in [23]. |
|  | MRI | 8 | 1357 | [22, 24-30] |
|  | Ultrasound | 13 | 3978 | [31-43] |
|  | CBCT | 8 | 904 | [44-51] |
|  | CT | 15 | 2746 | [52-66] |
|  | Cephalogram | 25 | 4162 | [67-88]; Köstler (1940), Weining (1958), Weiber (1940) in [31]. |
| 2018 T-Table | Needle Puncture | 23 | 951 | [1-15, 17-22]; Fisher and Moorman (year unknown) in [23]. |
|  | MRI | 6 | 1039 | [22, 24-28] |
|  | Ultrasound | 7 | 3536 | [31-36, 39] |
|  | CBCT | 1 | 100 | [44] |
|  | CT | 9 | 1720 | [52, 54, 56-61, 63] |
|  | Cephalogram | 14 | 1715 | [67-74, 82, 87, 88]; Köstler (1940), Weining (1958), Weiber (1940) in [31]. |
| New Studies | Needle Puncture | 1 | 100 | [16] |
|  | MRI | 2 | 318 | [29, 30] |
|  | Ultrasound | 6 | 442 | [37, 38, 40-43] |
|  | CBCT | 7 | 804 | [45-51] |
|  | CT | 6 | 1026 | [53, 55, 62, 64-66] |
|  | Cephalogram | 11 | 2447 | [75-81, 83-86] |

MRI = magnetic resonance imaging, CT = computed tomography, CBCT = cone beam computed tomography. The pooled n represents of the mean sample size across the five median landmarks.

**Table S2** Studies and sample sizes used to generate Fig. 5 & Fig. 6

|  | **Measurement Method** | **No. Studies** | **Pooled n** | **References** |
| --- | --- | --- | --- | --- |
| 2023 T-Table | Needle Puncture | 22 | 889 | [1-7, 9, 14-20, 22, 89-93]; Fisher and Moorman (year unknown) in [23]. |
|  | MRI | 4 | 577 | [22, 24, 26, 27] |
|  | Ultrasound | 11 | 1781 | [31, 35-43, 87] |
|  | CBCT | 8 | 1244 | [44-50, 94] |
|  | CT | 14 | 2268 | [52-54, 56-61, 63-65, 95, 96] |
| 2018 T-Table | Needle Puncture | 19 | 717 | [1-7, 9, 14, 15, 17-20, 22, 89-91]; Fisher and Moorman (year unknown) in [23]. |
|  | MRI | 4 | 577 | [22, 24, 26, 27] |
|  | Ultrasound | 5 | 1337 | [31, 35, 36, 39, 87] |
|  | CBCT | 1 | 100 | [44] |
|  | CT | 10 | 1721 | [52, 54, 56-61, 63, 96] |
| New Studies | Needle Puncture | 3 | 172 | [16, 92, 93] |
|  | MRI | 0 | - | - |
|  | Ultrasound | 6 | 444 | [37, 38, 40-43] |
|  | CBCT | 7 | 1144 | [45-50, 94] |
|  | CT | 4 | 548 | [53, 64, 65, 95] |

MRI = magnetic resonance imaging, CT = computed tomography, CBCT = cone beam computed tomography. The pooled n represents the mean sample size across the four bilateral landmarks.

**References**

1. Kollmann J, Büchly W (1898) Die Persistenz der Rassen und die Reconstruction der Physiognomie prähistorischer Schädel. Archiv für Anthropologie 25: 329-59.

2. Fischer E (1905) Anatomische Untersuchungen an den Kopfweichteilen zweier Papua. CorrBLAnthrop Ges Jhg 36: 118-22.

3. Birkner F (1904) Beiträge zur Rassenanatomie der Gesichtsweichteile. Corr Bl Anthrop Ges Jhg 34: 163-5.

4. Eggeling Hv (1909) Anatomische Untersuchungen an den Köpfen von ver Hereros, einem Herero- und einem Hottentottenkind. In: Schultze L, ed. Forschungsreise im westlichen und zentralen Südafrika. Denkschriften Jena. pp. 323-48.

5. Stadtmüller F (1922) Zur Beurteilung der plastischen Rekonstruktionsmethode der Physiognomie auf dem Schädel. Zeitschrift für Morpholologie und Anthropologie 22: 337-72.

6. Burkitt AN, Lightoller GHS (1923) Preliminary observations on the nose of the Australian aboriginal with a table of aboriginal head measurements. J Anat 57: 295-312.

7. Suzuki H (1948) On the thickness of the soft parts of the Japanese face. Journal of the Anthropological Society of Nippon 60: 7-11.

8. Forrest AS (1985) An investigation into the relationship between facial soft tissue thickness and age in Australian Caucasion cadavers. Thesis, The University of Queensland

9. O'Grady JF, Taylor RG, Clement JG. (1990) Facial tissue thickness: a study of cadavers in Melbourne. International Association of Forensic Science Scientific Symposium Adelaide.

10. Anderson W (1996) The correlation between soft tissue thickness and bony proportions of the skull and how they relate to facial reconstruction. Thesis, The University of Adelaide

11. Simpson E, Henneberg M (2002) Variation in soft-tissue thicknesses on the human face and their relation to craniometric dimensions. Am J Phys Anthropol 118: 121-33. <https://doi.org/10.1002/ajpa.10073>

12. Sutisno M (2003) Human facial soft-tissue thickness and its value in forensic facial reconstruction. Thesis, The University of Sydney

13. Domaracki M, Stephan CN (2006) Facial soft tissue thicknesses in Australian adult cadavers. J Forensic Sci 51: 5-10. <https://doi.org/10.1111/j.1556-4029.2005.00009.x>

14. Codinha S (2009) Facial soft tissue thicknesses for the Portuguese adult population. Forensic Sci Int 184: 80.e1-.e7. <https://doi.org/10.1016/j.forsciint.2008.11.011>

15. Tedeschi-Oliveira SV, Melani RFH, de Almeida N, de Paiva LA (2009) Facial soft tissue thickness of Braziallian adults. Forensic Sci Int 193: 127.e1-7. <https://doi.org/10.1016/j.forsciint.2009.09.002>

16. Navic P, Sinthubua A, Prasitwattanaseree S, Mahakkanukrauh P (2021) Facial soft tissue thickness for a Thai population using Needle puncture technique application for forensic facial reconstruction. Int Med J 28: 552-7.

17. Rhine JS, Campbell HR (1980) Thickness of facial tissues in American blacks. J Forensic Sci 25: 847-58. <https://doi.org/10.1520/JFS11301J>

18. Rhine JS, Moore CE (1984) Tables of facial tissue thickness of American Caucasoids in forensic anthropology. Maxwell Museum Technical Series 1:

19. Rhine S (1983) Tissue thickness for Southwestern Indians. Thesis, University of New Mexico

20. de Almeida NH, Michel-Crosato E, de Paiva LA, Biazevic MG (2013) Facial soft tissue thickness in the Brazilian population: new reference data and anatomical landmarks. Forensic Sci Int 231: 404.e1-7. <https://doi.org/10.1016/j.forsciint.2013.05.024>

21. Gerasimov MM (1955) Vosstanovlenie lica po cerepu. Izdat. Akademii Nauk SSSR Moskva.

22. Blythe T (1996) A re-assessment of the Rhine and Moore Technique in Forensic Facial Reconstruction. Thesis, The University of Manchester

23. Martin R, Saller K (1957) Lehrbuch der Anthropologie. Gustav Fischer Verlag Stuttgart.

24. Sahni D, Jit I, Gupta M, Singh P, Suri S. (2002) Preliminary study on facial soft tissue thickness by magnetic resonance imaging in Northwest Indians. Forensic Science Communications. <https://archives.fbi.gov/archives/about-us/lab/forensic-science-communications/fsc/jan2002/sahni.htm>. 1 January 2023

25. Sahni D, Sanjeev, Singh D, Jit I, Singh P (2008) Facial soft tissue thickness in northwest Indian adults. Forensic Sci Int 176: 137-46. <https://doi.org/10.1016/j.forsciint.2007.07.012>

26. Niinimaki S, Karttunen A. (2006) Finnish facial tissue thickness study. In: Herva V-P, ed. Proceedings of the 22nd Nordic Archaeological Conference. Gummerus Kirjapaino Oy University of Oulu. pp. 343-52.

27. Chen F, Chen Y, Yu Y, Qiang Y, Liu M, Fulton D (2011) Age and sex related measurement of craniofacial soft tissue thickness and nasal profile in the Chinese population. Forensic Sci Int 212: 272.e1-.e6. <https://doi.org/10.1016/j.forsciint.2011.05.027>

28. Sipahioglu S, Ulubay H, Diren HB (2012) Midline facial soft tissue thickness database of Turkish population: MRI study. Forensic Sci Int 219: 282.e1-.e38. <https://doi.org/10.1016/j.forsciint.2011.11.017>

29. Sandamini H, Jayawardena A, Batuwitage L et al (2018) Facial soft tissue thickness trends for selected age groups of Sri Lankan adult population. Forensic Sci Int 293: 102.e1-.e11. <https://doi.org/10.1016/j.forsciint.2018.10.001>

30. Eftekhari-Moghadam AR, Latifi SM, Nazifi HR, Rezaian J (2020) Influence of sex and body mass index on facial soft tissue thickness measurements in an adult population of southwest of Iran. Surg Radiol Anat 42: 627-33. <https://doi.org/10.1007/s00276-019-02409-2>

31. Helmer R (1984) Schädelidentifizierung durch elekroniesche Bildmischung: Zugleich ein Beitrag zur Konstitutionsbiometrie und Dickenmessung der Gesichtsweichteile. Krminalistik-Verlag Heidelberg.

32. Lebedinskaya GV, Balueva TS, Veselovskaya EV (1993) Principles of facial reconstruction. In: İşcan MY, Helmer RP, eds. Forensic Analysis of the Skull. Wiley-Liss New York. pp. 183-98.

33. Lebedinskaya GV, Veselovskaya EV (1986) Ultrasonic measurements of the thickness of soft facial tissue among the Bashkirs. Annales Academiae Scientiarium Fennicae SerA 5 Medica 175: 91-5.

34. Manhein MH, Listi GA, Barsley RE, Musselman R, Barrow NE, Ubelaker DH (2000) In vivo facial tissue depth measurements for children and adults. J Forensic Sci 45: 48-60.

35. De Greef S, Claes P, Vandermeulen D, Mollemans W, Suetens P, Willems G (2006) Large-scale *in-vivo* Caucasian soft tissue thickness database for craniofacial reconstruction. Forensic Sci Int 159S: S126-S46. <https://doi.org/10.1016/j.forsciint.2006.02.034>

36. Chan WN, Listi GA, Manhein MH (2011) *In vivo* facial tissue depth study of Chinese-American adults in New York City. J Forensic Sci 56: 350-8. <https://doi.org/10.1111/j.1556-4029.2010.01640.x>

37. MacNeil JAB, Peckmann TR, Mussett M (2013) Asymmetry in forensic three-dimensional facial reconstruction: An assessment of facial asymmetry in adult First Nations Nova Scotian facial soft tissue depth data. Canadian Society of Forensic Sciences Journal 46: 141-65. <https://doi.org/10.1080/00085030.2013.10773759>

38. Peckmann TR, Harris M, Huculak M, Pringle A, Fournier M (2015) In vivo facial tissue depth for Canadian Mi'kmaq adults: A case study from Nova Scotia, Canada. J Forensic Leg Med 29: 43-53. <https://doi.org/10.1016/j.jflm.2014.12.004>

39. Jia L, Qi B, Yang J, Zhang W, Lu Y, Zhang H-L (2016) Ultrasonic measurement of facial tissue depth in a Northern Chinese Han population. Forensic Sci Int 259: 247.e1-.e6. <https://doi.org/10.1016/j.forsciint.2015.12.012>

40. Stephan CN, Priesler R, Bulut O, Bennett MB (2016) Turning the tables of sex distinction in craniofacial identification: why females possess thicker facial soft tissues than males, not vice versa. Am J Phys Anthropol 161: 283-95. <https://doi.org/10.1002/ajpa.23029>

41. Stephan CN, Preisler R (2018) *In vivo* facial soft tissue thicknesses of adult Australians. Forensic Sci Int 282: 220.e1-.e12. <https://doi.org/10.1016/j.forsciint.2017.11.014>

42. Stephan CN, Sievwright E (2018) Facial soft tissue thickness (FSTT) estimation models—and the strength of correlations between craniometric dimensions and FSTTs. Forensic Sci Int 286: 128-40. <https://doi.org/10.1016/j.forsciint.2018.03.011>

43. Kimura Y, Okazaki K (2018) Facial soft tissue depth measured using ultrasonography: Towards facial approximation for Japanese crania. Anthropol Sci (Japanese Series) 126: 37-54. <http://doi.org/10.1537/asj.180420>

44. Hwang H-S, Park M-K, Lee W-J, Cho J-H, Kim B-K, Wilkinson CM (2012) Facial soft tissue thickness database for craniofacial reconstruction in Korean adults. J Forensic Sci 57: 1442-7. <https://doi.org/10.1111/j.1556-4029.2012.02192.x>

45. Meundi MA, David CM (2019) Application of cone beam computed tomography in facial soft tissue thickness measurements for craniofacial reconstruction. J Oral Maxillofac Pathol 23: 114-21. <https://doi.org/10.4103/jomfp.JOMFP_20_19>

46. Meundi MA, David CM (2019) Morphometric analysis of facial soft tissue thickness for sexual dimorphism: A cone beam computed tomography study. Int J Forensic Med Toxicol Sci 4: 60-7. <http://doi.org/10.18231/j.ijfmts.2019.014>

47. Meundi MA, David CM (2019) Facial soft tissue thickness in South Indian adults with varied occlusions–A cone beam computed tomography study. J Indian Acad Oral Med Radiol 31: 194-202. <https://doi.org/10.4103/jiaomr.jiaomr_83_19>

48. Beaini TL, Miamoto P, Duailibi-Neto EF, Tedeschi-Oliveira SV, Chilvarquer I, Melani RFH (2021) Facial soft tissue depth measurements in cone-beam computed tomography: A study of a Brazilian sample. Leg Med 50: 1-13. <https://doi.org/10.1016/j.legalmed.2021.101866>

49. de Barros F, da Costa Serra M, Kuhnen B, Scarso Filho J, Gonçalves M, Fernandes CMS (2021) Midsagittal and bilateral facial soft tissue thickness: a Cone-Beam Computed Tomography assessment of Brazilian living adults. Forensic Imaging 25: 1-8. <https://doi.org/10.1016/j.fri.2021.200444>

50. Moritsugui DS, Fugiwara FVG, Vassallo FNS, Mazzilli LEN, Beaini TL, Melani RFH (2022) Facial soft tissue thickness in forensic facial reconstruction: Impact of regional differences in Brazil. PloS ONE 17: 1-15. <https://doi.org/10.1371/journal.pone.0270980>

51. Shehata TI, Khattab N, Wahab TMA, Ekram AM, Elbashar YH (2022) Imaging analysis of cone beam computed tomography for present Egyptians facial soft tissue thicknesses. J Opt 52: 915-23. <https://doi.org/10.1007/s12596-022-00934-9>

52. Phillips VM, Smuts NA (1996) Facial reconstruction: Utilization of computerized tomography to measure facial tissue thickness in a mixed racial population. Forensic Sci Int 83: 51-9. <https://doi.org/10.1016/0379-0738(96)02010-5>

53. Bellmann D, Fuchs T, Weidenbusch A et al (2007) Computer-aided measurement of the tissue thickness of deceased persons with computer tomography scans of the head. In: Buzug TM, Sigl KM, Bongartz J, Prüfer K, eds. Facial Reconstruction: Forensic, Medical and Archeological Methods of the Reconstruction of Soft Facial Parts Gesichtsrekonstruktion: Forensische, Medizinische Und Archäologische Methoden Der Gesichtsweichteilrekonstruktion Luchterhand Munich. pp. 21-39.

54. Tilotta F, Richard F, Glaunes J et al (2009) Construction and analysis of a head CT-scan database for craniofacial reconstruction. Forensic Sci Int 191: 112.e1-12. <https://doi.org/10.1016/j.forsciint.2009.06.017>

55. Shimofusa R, Yamamoto S, Horikoshi T, Yokota H, Iwase H (2009) Applicability of facial soft tissue thickness measurements in 3-dimensionally reconstructed multidetector-row CT images for forensic anthropological examination. Leg Med 11: S256-S9. <https://doi.org/10.1016/j.legalmed.2009.01.036>

56. Cavanagh D, Steyn M (2011) Facial reconstruction: Soft tissue thickness values for South African black females. Forensic Sci Int 206: 215.e1-.e7. <https://doi.org/10.1016/j.forsciint.2011.01.009>

57. Dong Y, Huang L, Feng Z, Bai S, Wu G, Zhao Y (2012) Influence of sex and body mass index on facial soft tissue thickness measurements of the northern Chinese adult population. Forensic Sci Int 222: 396.e1-.e7. <https://doi.org/10.1016/j.forsciint.2012.06.004>

58. Guyomarc'h P, Santos F, Dutailly B, Coqueugniot H (2013) Facial soft tissue depths in French adults: variability, specificity and estimation. Forensic Sci Int 231: 411.e1-.e10. <https://doi.org/10.1016/j.forsciint.2013.04.007>

59. Bulut O, Sipahioglu S, Hekimoglu B (2014) Facial soft tissue thickness database for craniofacial reconstruction in the Turkish adult population. Forensic Sci Int 242: 44-61. <https://doi.org/10.1016/j.forsciint.2014.06.012>

60. Parks CL, Richard AH, Monson KL (2014) Preliminary assessment of facial soft tissue thickness utilizing three-dimensional computed tomography models of living individuals. Forensic Sci Int 237: 146.e1-.e10. <https://doi.org/10.1016/j.forsciint.2013.12.043>

61. Chung JH, Hsu HT, Chen HT, Huang GS, Shaw KP (2015) A CT-scan database for the facial soft tissue thickness of Taiwan adults. Forensic Sci Int 253: 132.e1-.e11. <https://doi.org/10.1016/j.forsciint.2015.04.028>

62. Lodha A, Mehta M, Patel M, Menon SK (2016) Facial soft tissue thickness database of Gujarati population for forensic craniofacial reconstruction. Egypt J Forensic Sci 6: 126-34. <https://doi.org/10.1016/j.ejfs.2016.05.010>

63. Drgáčová A, Dupej J, Velemínská J (2016) Facial soft tissue thicknesses in the present Czech population. Forensic Sci Int 260: 106.e1-.e17. <https://doi.org/10.1016/j.forsciint.2016.01.011>

64. Tanaka C, Utsuno H, Makino Y et al (2020) Facial soft tissue thickness of the Japanese population determined using post mortem computed tomography images. Forensic Imaging 23: 1-7. <https://doi.org/10.1016/j.fri.2020.200423>

65. Wang D, Zhang Q, Zeng N, Wu Y (2022) Age-Related Changes in Facial Soft Tissue of Han Chinese: A Computed Tomographic Study. Dermatol Surg 48: 741-6. <https://doi.org/10.1097/dss.0000000000003460>

66. Cha K (2013) Soft-tissue thickness of South Korean adults with normal facial profiles. Korean J Orthod 43: 178-85. <https://doi.org/10.4041/kjod.2013.43.4.178>

67. Edelman H (1938) Die Profilanalyse: Eine Studie an photographischen und röntgenographischen Durchdringungsbildern. Zeitschrift fur Morpholologie und Anthropologie 37: 166-88.

68. Ogawa H (1960) Anatomical study on the Japanese head by X-ray cephalometry. The Journal of the Tokyo Dental College Society [Shika Gakuho] 60: 17-34.

69. Helwin H (1969) Die Profilanalyse, eine Möglichkeit der Identifizierung unbekannter Schädel. Gegenbaurs Morphologisches Jahrbuch 113: 467-99.

70. Dumont ER (1986) Mid-facial tissue depths of white children: an aid in facial feature reconstruction. J Forensic Sci 31: 1463-9.

71. George RM (1987) The lateral craniographic method of facial reconstruction. J Forensic Sci 32: 1305-30.

72. Miyasaka S (1999) Progress in facial reconstruction technology. Forensic Science Review 11: 50-90.

73. Garlie TN, Saunders SR (1999) Midline facial tissue thicknesses of subadults from a longitudinal radiographic study. J Forensic Sci 44: 61-7.

74. Kurkcuoglu A, Pelin C, Ozener B, Zagyapan R, Sahinoglu Z, Yazici AC (2011) Facial soft tissue thickness in individuals with different occlusion patterns in adult Turkish subjects. Homo 62: 288-97. <https://doi.org/10.1016/j.jchb.2011.06.001>

75. Kamak H, Celikoglu M (2012) Facial soft tissue thickness among skeletal malocclusions: is there a difference? Korean J Orthod 42: 23-31. <https://doi.org/10.4041/kjod.2012.42.1.23>

76. Utsuno H, Kageyama T, Keiichi U, Kibayashi K (2014) Facial soft tissue thickness differences among three skeletal classes in Japanese population. Forensic Sci Int 236: 175-80. <https://doi.org/10.1016/j.forsciint.2013.12.040>

77. Gungor K, Bulut O, Hizliol I, Hekimoglu B, Gurcan S (2015) Variations of midline facial soft tissue thicknesses among three skeletal classes in Central Anatolian adults. Leg Med 17: 459-66. <https://doi.org/10.1016/j.legalmed.2015.09.001>

78. Jeelani W, Fida M, Shaikh A (2015) Facial soft tissue thickness among various vertical facial patterns in adult Pakistani subjects. Forensic Sci Int 257: 517.e1-.e6. <https://doi.org/10.1016/j.forsciint.2015.09.006>

79. Jeelani W, Fida M, Shaikh A (2015) Facial soft tissue thickness among three skeletal classes in adult Pakistani subjects. J Forensic Sci 60: 1420-5. <https://doi.org/10.1111/1556-4029.12851>

80. Hamid S, Abuaffan AH (2016) Facial soft tissue thickness in a sample of Sudanese adults with different occlusions. Forensic Sci Int 266: 209-14. <https://doi.org/10.1016/j.forsciint.2016.05.018>

81. Kotrashetti VS, Mallapur MD (2016) Radiographic assessment of facial soft tissue thickness in South Indian population - An anthropologic study. J Forensic Leg Med 39: 161-8. <https://doi.org/10.1016/j.jflm.2016.01.032>

82. Wang J, Zhao X, Mi C, Raza I (2016) The study on facial soft tissue thickness using Han population in Xinjiang. Forensic Sci Int 266: 585.e1-.e5. <https://doi.org/10.1016/j.forsciint.2016.04.032>

83. Ayoub F, Saadeh M, Rouhana G, Haddad R (2019) Midsagittal facial soft tissue thickness norms in an adult mediterranean population. Forensic Sci Int 294: 217.e1-.e7. <https://doi.org/10.1016/j.forsciint.2018.10.021>

84. Chu G, Han M, Ji L et al (2020) Will different sagittal and vertical skeletal types relate the soft tissue thickness: A study in Chinese female adults. Leg Med 42: 1-8. <https://doi.org/10.1016/j.legalmed.2019.101633>

85. Kunnath JT, Subrahmanya RM, Dhillon H (2020) Assessment of Facial Soft Tissue Thickness in Individuals having Skeletal Class II Malocclusion. World J Dent 11: 179-84. <https://doi.org/10.5005/jp-journals-10015-1724>

86. Sarilita E, Rynn C, Mossey PA, Black S, Oscandar F (2020) Facial average soft tissue depth variation based on skeletal classes in Indonesian adult population: A retrospective lateral cephalometric study. Leg Med 43: 1-7. <https://doi.org/10.1016/j.legalmed.2019.101665>

87. Aulsebrook WA, Becker PJ, İşcan MY (1996) Facial soft-tissue thickness in the adult male Zulu. Forensic Sci Int 79: 83-102. <https://doi.org/10.1016/0379-0738(96)01893-2>

88. Leopold D (1968) Identifikation durch Schädeluntersuchung unter besonderer Berücksichtigung der Superprojektion. Thesis, Karl-Marx-Universität

89. His W (1895) Anatomische Forschungen über Johann Sebastian Bach's Gebeine und Antlitz nebst Bemerkungen über dessen Bilder. Abh MathPhysikal KI Kgl Sachs Ges Wiss 22: 379-420.

90. Stewart TD (1954) Evaluation of evidence from the skeleton. In: Gradwohl RBH, ed. Leg Med. C. V. Mosby St. Louis. pp. 407-50.

91. Suazo GIC, Cantín LM, Zavando MDA, Perez RFJ, Torres MSR (2008) Comparisons in soft-tissue thicknesses on the human face in fresh and embalmed corpses using needle puncture method. Int J Morphol 26: 165-9. <http://dx.doi.org/10.4067/S0717-95022008000100027>

92. Salazar CB, Matamala DZ, Cantín M, Galdames IS (2010) Facial tissue thickness in Chilean cadavers with medico-legal purposes. Int J Odontostomat 4: 215-22. <http://dx.doi.org/10.4067/S0718-381X2010000300002>

93. Torres Muñoz SR, Cantín M, Pérez Rojas FJ, Suazo Galdames I (2011) Evaluation of facial asymmetry using soft-tissue thickness for forensic purposes. Int J Morphol 29: 1033-9. <http://dx.doi.org/10.4067/S0717-95022011000300063>

94. Deng C, Wang D, Chen J et al (2020) Facial soft tissue thickness in Yangtze River delta Han population: Accurate assessment and comparative analysis utilizing Cone-Beam CT. Leg Med 44: 1-12. <https://doi.org/10.1016/j.legalmed.2020.101693>

95. Toneva D, Nikolova S, Georgiev I et al (2018) Facial soft tissue thicknesses in Bulgarian adults: relation to sex, body mass index and bilateral asymmetry. Folia Morphol 77: 570-82. <https://doi.org/10.5603/FM.a2017.0114>

96. Perlaza Ruiz N (2013) Facial soft tissue thickness of Colombian adults. Forensic Sci Int 229: 160.e1-.e9. <https://doi.org/10.1016/j.forsciint.2013.03.017>
